# PfGSTF2 endows resistance to quizalofop‐p‐ethyl in *Polypogon fugax* by GSH conjugation

**DOI:** 10.1111/pbi.14491

**Published:** 2024-10-28

**Authors:** Wen Chen, Dingyi Bai, Yuxi Liao, Qin Yu, Lianyang Bai, Lang Pan

**Affiliations:** ^1^ College of Plant Protection Hunan Agricultural University Changsha China; ^2^ College of Agriculture Tarim University Alaer China; ^3^ Australian Herbicide Resistance Initiative (AHRI), School of Agriculture and Environment University of Western Australia Crawley Western Australia Australia

**Keywords:** *Polypogon fugax*, glutathione *S*‐transferases, weed resistance, herbicide metabolism

## Abstract

Populations of *Polypogon fugax* have developed resistance to many acetyl‐CoA carboxylase (ACCase)‐inhibiting herbicides. This resistance threats the effectiveness and sustainability of herbicide use. In our previous research, a field *P. fugax* population exhibited GST‐based metabolic resistance to the widely used ACCase‐inhibiting herbicide quizalofop‐p‐ethyl. Here, in this current study, we identified and characterized two GST genes (named as *PfGSTF2* and *PfGSTF58*) that showed higher expression levels in the resistant than the susceptible population. Transgenic rice calli overexpressing *PfGSTF2*, but not *PfGSTF58*, became resistant to quizalofop‐p‐ethyl and haloxyfop‐R‐methyl. This reflects similar cross‐resistance pattern to what was observed in the resistant *P. fugax* population. Transgenic rice seedlings overexpressing *PfGSTF2* also exhibited resistance to quizalofop‐p‐ethyl. In contrast, CRISPR/Cas9 knockout of the orthologue gene in rice seedlings increased their sensitivity to quizalofop‐p‐ethyl. LC–MS analysis of *in vitro* herbicide metabolism by *Escherichia coli*‐expressed recombinant PfGSTF2 revealed that quizalofop (but not haloxyfop) was detoxified at the ether bond, generating the GSH‐quizalofop conjugate and a propanoic acid derivative with greatly reduced herbicidal activity. Equally, these two metabolites accumulated at higher levels in the resistant population than the susceptible population. In addition, both recombinant PfGSTF2 and PfGSTF58 can attenuate cytotoxicity by reactive oxygen species (ROS), suggesting a role in plant defence against ROS generated by herbicides. Furthermore, the GST inhibitor (NBD‐Cl) reversed resistance in the resistant population, and PfGSTF2 (but not PfGSTF58) responded to NBD‐Cl inhibition. All these suggest that PfGSTF2 plays a significant role in the evolution of quizalofop resistance through enhanced herbicide metabolism in *P. fugax*.

## Introduction

Widespread herbicide resistance in crop weed species is a major threat to grain production and global food security. Given the urgent need to reduce pesticide usage while addressing rising food demands, it is important to elucidate the mechanisms underlying herbicide resistance, especially non‐target‐site resistance (NTSR). NTSR constitutes less explored but increasingly concerning mechanism in herbicide resistance (Gaines *et al*., 2020; Han *et al*., 2021). Weeds with NTSR often exhibit alterations in herbicide uptake, translocation and metabolism (Gaines *et al*., 2020; Powles and Yu, 2010). Among these NTSR mechanisms, enhanced herbicide metabolism (i.e. metabolic herbicide resistance) exerts broader impact on resistance spectrum: weeds may become resistant to herbicides of multiple modes of action, even new or newly registered herbicides, rendering weed control challenging (Yu and Powles, 2014; Gao *et al*., 2023). Two major enzyme families, cytochrome P450 monooxygenases (P450s) and glutathione *S*‐transferases (GSTs), are involved in metabolic herbicide resistance (Dimaano and Iwakami, 2021; Iwakami *et al*., 2019; Powles and Yu, 2010).

GSTs are essential metabolic enzymes present in bacteria, fungi, animals and plants (Bela *et al*., 2015; Edwards *et al*., 2000; Reade *et al*., 2004). GSTs play a key role in phase II detoxification of xenobiotics by conjugating reduced glutathione (GSH) with electrophilic compounds (Concepcion *et al*., 2021; Cummins *et al*., 2013). Plant GSTs have been known to be important mediators of metabolic resistance to certain herbicides (Cummins *et al*., 2013; Edwards *et al*., 2000; Powles and Yu, 2010; Reade *et al*., 2004). In land plants, the phi‐ and tau‐classes of GSTs have been identified as key contributors to herbicide tolerance. At least 29 GSTs have been identified in crop species (e.g. maize, rice, wheat and soybean) to be involved in herbicide detoxification, although most have only been validated *in vitro* (Casey and Dolan, 2023). For instance, laboratory‐generated GST transgenic crops can tolerate sulfonylurea (Hu *et al*., 2009), α‐chloroacetamide, thiocarbamate and/or diphenyl ether herbicides (Benekos *et al*., 2010; Karavangeli *et al*., 2005; Milligan *et al*., 2001; Skipsey *et al*., 2005).

GSTs from weedy species also play important roles in metabolic herbicide resistance (Reade *et al*., 2004). For example, early research by Gronwald *et al*. (1989) and Anderson and Gronwald (1991) found higher GST activities led to strong resistance to triazine herbicides in velvetleaf (*Abutilon theophrasti*) by GSH‐triazine conjugation. Bakkali *et al*. (2007) and Ducker *et al*. (2019) reported that increased GST activities were related to flufenacet resistance in *Lolium* spp and fenoxaprop‐p‐ethyl resistance in *Echinochloa phyllopogon*. In *L. rigidum*, GST‐mediated pyroxasulfone conjugation is crucial for the resistance to this herbicide (Goggin *et al*., 2021, 2024). Concepcion *et al*. (2021) identified novel detoxification mechanism in *Amaranthus tuberculatus* for resistance to a non‐commercial HPPD‐inhibiting herbicide, and this mechanism involves Phase I reduction–dehydration followed by Phase II GSH conjugation. Evans Jr. *et al*. (2017) indicated constitutive expression of *AtuGSTF2* may contribute to atrazine resistance in *A. tuberculatus*. Work by Georgakis *et al*. (2020) and Schwarz *et al*. (2021) elucidated the structure and function of phi‐class GSTs in weeds. Stafford (2017) and Lowe *et al*. (2024) developed molecular GST biomarkers to diagnosis metabolic resistance in *Alopecurus myosuroides*.

Weed genome research has shown that GST evolution is complex. Massive gene duplication and directed evolution of *GST* genes are likely key factors driving the evolution of multiple‐herbicide resistance in grass weed species (Ducker *et al*., 2024; Ioannou *et al*., 2022; Parcharidou *et al*., 2024). However, limited progress has been made in cloning and characterizing *GST* genes that confer herbicide resistance in weedy plant species, although it has been implicated that overexpression of *GST* gene contributes to weed resistance to certain herbicides (Bai *et al*., 2020; Chen *et al*., 2020). For example, Cummins *et al*. (2013) revealed a phi‐class GST, *AmGSTF1*, conferred resistance to multiple herbicides in *A. myosuroides*; Liu *et al*. (2023) established a tau‐class GST, *GSTU1*, endowed resistance to haloxyfop‐P‐methyl in *Digitaria sanguinalis* in transgenic plants. Parcharidou *et al*. (2023) characterized three tau‐ and two phi‐class recombinant GSTs in flufenacet‐resistant *A. myosuroides* that can detoxify flufenacet and other very‐long‐chain fatty acids (VLCFA)‐inhibiting herbicides *in vitro*.

Asia minor bluegrass (*Polypogon fugax*) is a major crop weed in winter canola and wheat fields and causes significant declines in crop yields (Chen *et al*., 2020; Zhao *et al*., 2019). For nearly two decades, quizalofop‐p‐ethyl, an acetyl‐CoA carboxylase (ACCase)‐inhibiting herbicide, has been commonly used for *P. fugax* control. However, in 2014, Tang *et al*. (2014) reported the first case of quizalofop‐p‐ethyl resistance in *P. fugax* population in China. Now, *P. fugax* has evolved resistance to at least 10 ACCase‐ and acetolactate synthase (ALS)‐inhibiting herbicides (Chen *et al*., 2020; Mo *et al*., 2022; Yu *et al*., 2021; Zhao *et al*., 2019). NTSR to herbicides has been increasingly implicated in *P. fugax* (Chen *et al*., 2023; Zhao *et al*., 2022; Zhou *et al*., 2022); however, the key NTSR genes in *P. fugax* remain unknown. Our previous study investigating quizalofop‐p‐ethyl resistance in *P. fugax* showed that resistance did not involve ACCase gene mutations but rather could be reversed by the GSTs inhibitor 4‐chloro‐7‐nitrobenzoxadiazole (NBD‐Cl) (Chen *et al*., 2020). Moreover, there was an increase in GST enzyme activity and overexpression of the two GST genes in the resistant population following herbicide treatment (Chen *et al*., 2020). These findings indicate that the resistance mechanism of the *P. fugax* population may exhibit GST‐based NTSR. Here, we clone and functionally characterize *GST* genes that confer resistance to quizalofop‐p‐ethyl in *P. fugax*. These findings provide fundamental understanding of GST‐mediated metabolic herbicide resistance in weeds.

## Results

### Enhanced quizalofop acid metabolism in plants of the resistant (SC‐R) *P. fugax* population

**Figure 1:**
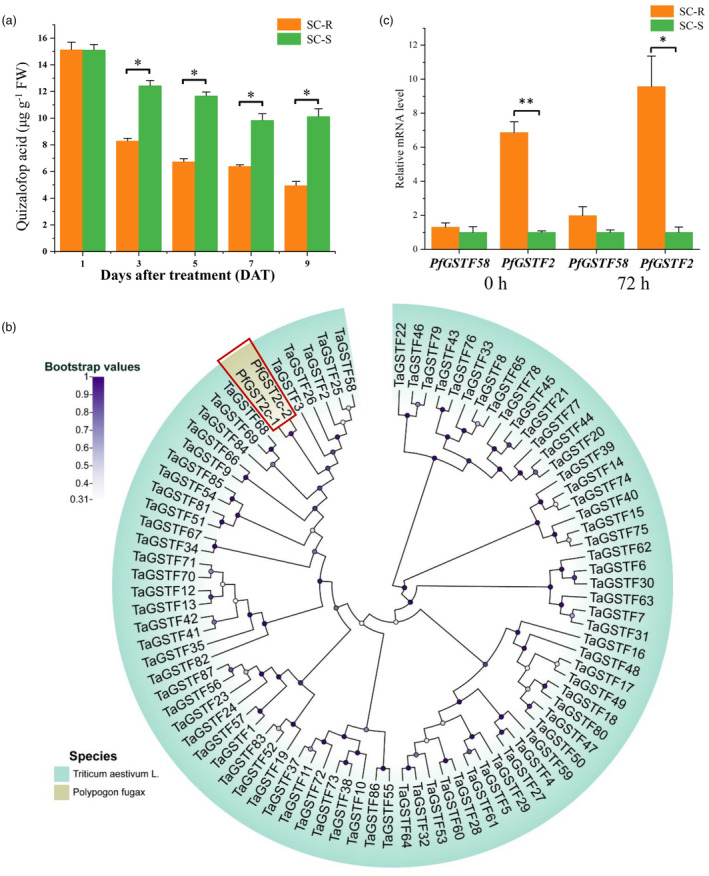
Characterization of quizalofop acid metabolism and GST genes in SC‐R and SC‐S *P. fugax* populations. (a) UHPLC analysis of tissue quizalofop acid levels in SC‐R and SC‐S *P. fugax* plants. (b) Phylogenetic relationships between *PfGST2c* (*PfGSTF2*) and wheat phi‐classes GSTs. (c) Expression levels of *PfGSTF2* and *PfGSTF58* in SC‐R and SC‐S populations, 0 and 72 h after quizalofop‐p‐ethyl treatment. Data in (a) and (c) are means ± SE (*n* = 3). * and ** indicate *P* < 0.05 and 0.01, respectively, by Student's *t*‐test.

Quizalofop acid is the active form of quizalofop‐p‐ethyl, and thus, the level of quizalofop acid was compared in plants of the quizalofop‐p‐ethyl‐resistant (SC‐R) and quizalofop‐p‐ethyl‐susceptible (SC‐S) populations. There was no significant difference in tissue quizalofop acid content between the SC‐R and SC‐S plants on day 1 after quizalofop‐p‐ethyl treatment (Figure 1a). However, from day 3 onwards the quizalofop acid level in SC‐R was about 2‐fold significantly (*P* < 0.05) lower than in SC‐S (Figure 1a). This result indicates that resistance to quizalofop‐p‐ethyl may be associated with enhanced herbicide metabolism.

### Full‐sequence cloning and analysis of the candidate *P. fugax* GST genes

Constitutive overexpression of the *GST2c* genes was associated with herbicide resistance in SC‐R *P. fugax* in our previous study (Chen *et al*., 2020). Ten *P. fugax GST2c*‐positive clones were sequenced from each of the three SC‐S and three SC‐R individual plants, and two *GST2c* transcripts (designated *PfGST2c‐1* and *PfGST2c‐2*) were obtained, encoding 219 amino acids. Nucleotide sequence alignment showed that *PfGST2c‐1* and *PfGST2c‐2* had 89.9% identity and the two genes are identical between SC‐S and SC‐R plants (Figure S1a). Deduced amino acid sequence alignment showed that PfGST2c‐1 and PfGST2c‐2 had 86.8% identity (Figure S1b). The phylogenetic tree analysis revealed a clear subdivision of the eight classes of wheat GSTs, *PfGST2c‐1* and *PfGST2c‐2* were placed in the phi‐class with strong bootstrap support (Figure S2a). In addition, the MEME online tool was used to identify the motif composition in PfGST2c, which is similar to other TaGSTFs possessing four conserved motifs (Figure S2b). Therefore, based on phylogenetic analysis and protein sequence identity, *PfGST2c‐1* and *PfGST2c‐2* should be classified as the phi‐class GSTs. *PfGST2c‐1* and *PfGST2c‐2* were placed in the same branch as *TaGSTF2*, *TaGSTF3*, *TaGSTF25*, *TaGSTF26* and *TaGSTF58* (Figure 1b). Among them, *PfGST2c‐1* had the highest amino acid identity with *TaGSTF58* (86.3%) and *PfGST2c‐2* with *TaGSTF2* (87.2%). Therefore, *PfGST2c‐1* and *PfGST2c‐2* were named *PfGSTF58* and *PfGSTF2*, respectively.

### *PfGSTF2* is higher expressed in SC‐R population

Expression of the two identified candidate GST genes *PfGSTF2* and *PfGSTF58* was compared in plants of the SC‐R and SC‐S populations. *PfGSTF58* was not differentially expressed (*P* > 0.05) in the two populations with and without the herbicide treatment, whereas *PfGSTF2* was consistently higher expressed (6.9‐ to 9.6‐fold) (*P* < 0.05) in the SC‐R population (Figure 1c). This suggests that overexpression of *PfGSTF2* may related to quizalofop‐p‐ethyl resistance in the SC‐R population.

### PfGSTF2 confers herbicide resistance in transgenic rice

**Figure 2:**
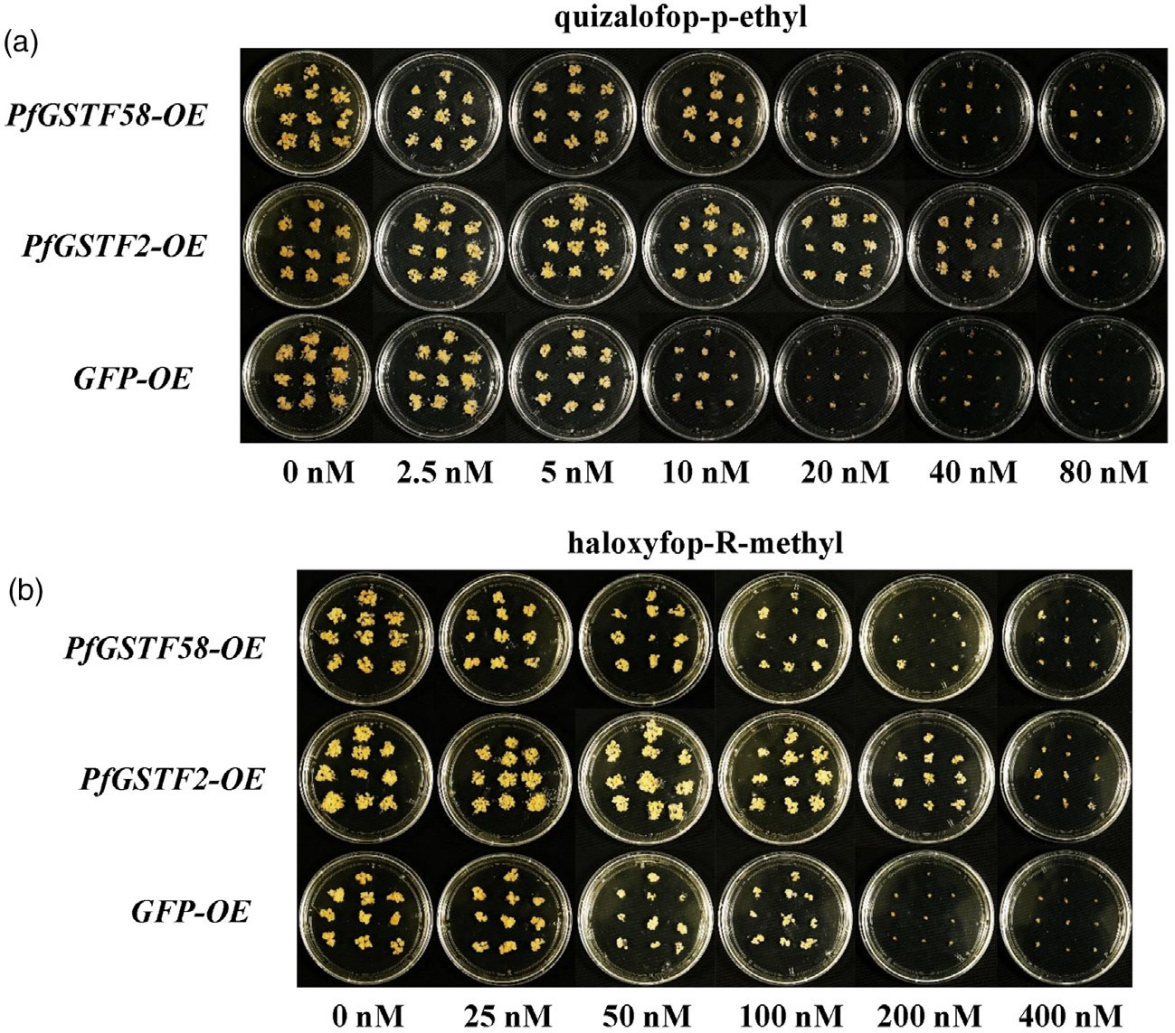
Herbicide sensitivity of the rice calli expressing *PfGSTF2* or *PfGSTF58* versus *GFP* control. Ten independent calli expressing *PfGSTF2*, *PfGSTF58* or *GFP* were selected with hygromycin and grown for 3 weeks on N6D medium containing (a) quizalofop‐p‐ethyl and (b) haloxyfop‐R‐methyl.

Proliferation of transgenic rice calli expressing *PfGSTF58* (*PfGSTF58*‐OE) was better than transgenic calli expressing *GFP* (*GFPGFP*‐OE), but weaker than transgenic rice calli expressing *PfGSTF2* (*PfGSTF2*‐OE) (Figure 2a). For example, *PfGSTF2*‐OE calli was able to grow at higher concentrations of haloxyfop‐R‐methyl (>100 nM) compared to *PfGSTF58* and *GFP*‐OE (50–100 nM) (Figure 2b). This indicates that overexpression of *PfGSTF2* (and to a less extent, *PfGSTF58*) confers resistance to quizalofop‐p‐ethyl in rice calli. In contrast, neither *PfGSTF2*‐OE nor *PfGSTF58*‐OE calli showed better growth than *GFP*‐OE under pinoxaden and fenoxaprop‐p‐ethyl treatments (Figure S3). Furthermore, transgenic rice seedlings (T_0_) overexpressing *PfGSTF2* survived at 26.3 g ha^−1^ quizalofop‐p‐ethyl; however, *GFP* control seedlings died (Figure S4). Results from transgenic calli and seedlings establish that overexpression of the *PfGSTF2* gene confers resistance in rice to quizalofop‐p‐ethyl and haloxyfop‐R‐methyl, and thus likely endows resistance to these herbicides in SC‐R population.

### CRISPR/Cas9 knockout of the rice orthologous *OsGSTF2* gene increases quizalofop‐p‐ethyl sensitivity in rice seedlings

The *OsGSTF2* gene, orthologous to *PfGSTF2*, was cloned from rice, and the knockout (KO) lines generated. A nucleotide insertion in *OsGSTF2* was detected in all homozygous T1 KO lines (Figure S5), causing a frame change that results in early transcription termination. Seedlings of a single T2 KO line (*osgstf2*) were examined for quizalofop‐p‐ethyl susceptibility. At 7 DAT with quizalofop‐p‐ethyl at 20 and 40 nM, untransformed wild type (WT) seedlings had reduction in shoot elongation by 37 ± 2.6% and 44 ± 3.2%, whereas the KO line had 44 ± 1.8% and 76 ± 5.2% reduction, respectively (Figure S6). Evidently, knockout of the rice orthologous *OsGSTF2* gene increased quizalofop‐p‐ethyl susceptibility of rice seedlings (Figure S6). This result provides evidence of reverse genetics for the role of *PfGSTF2* in quizalofop‐p‐ethyl resistance.

### Recombinant PfGSTF2 has a lower standard GST activity and can be inhibited by NBD‐Cl

Recombinant PfGSTF2 and PfGSTF58 was purified and examined for their kinetics properties. Preliminary experiments demonstrated that the highest yield of PfGSTF2 recombinant protein was obtained with the imidazole eluate at 200 mM (Figure S7a), so this condition was used for protein purification. PfGSTF2 and PfGSTF58 were purified to distinct bands between 25 and 35 kDa, and the molecular mass of the recombinant protein PfGSTF58 was slightly larger than that of PfGSTF2 (Figure S7a). The *Vmax* of PfGSTF58 towards the standard substrate 1‐chloro‐2,4‐dinitrobenzene (CDNB) was estimated to be 15.93 ± 0.94 μmol mg^−1^ min^−1^, which is 4.67‐fold (*P* < 0.05) greater than that of PfGSTF2 (Figure S7b, c). The *Km* (CDNB) of PfGSTF58 was 0.45 ± 0.09 mM, which is 3.47‐fold (*P* < 0.05) lower than that of PfGSTF2 (Figure S7b, c). These results indicate that PfGSTF2 has significantly lower activity and affinity for the standard substrate CDNB compared to PfGSTF58.

In addition, as the known GST inhibitor NBD‐Cl can reverse quizalofop‐p‐ethyl resistance in SC‐R population, the inhibitory effect of NBD‐Cl on PfGSTF2 and PfGSTF58 was also examined. No GST activity was recorded without NBD‐Cl, and maximum activity detected with NBD‐Cl at 0.1–0.3 mM (Figure S8). However, greatly reduced GST activity (6‐fold) was observed at NBD‐Cl >0.3 mM for PfGSTF2 (Figure S8). Clearly, *in vitro* NBD‐Cl acts as a substrate and incurs inhibitory effect more on PfGSTF2 (than on PfGSTF58) at higher concentrations.

### Recombinant PfGSTF2 can conjugate quizalofop acid *in vitro*

**Figure 3:**
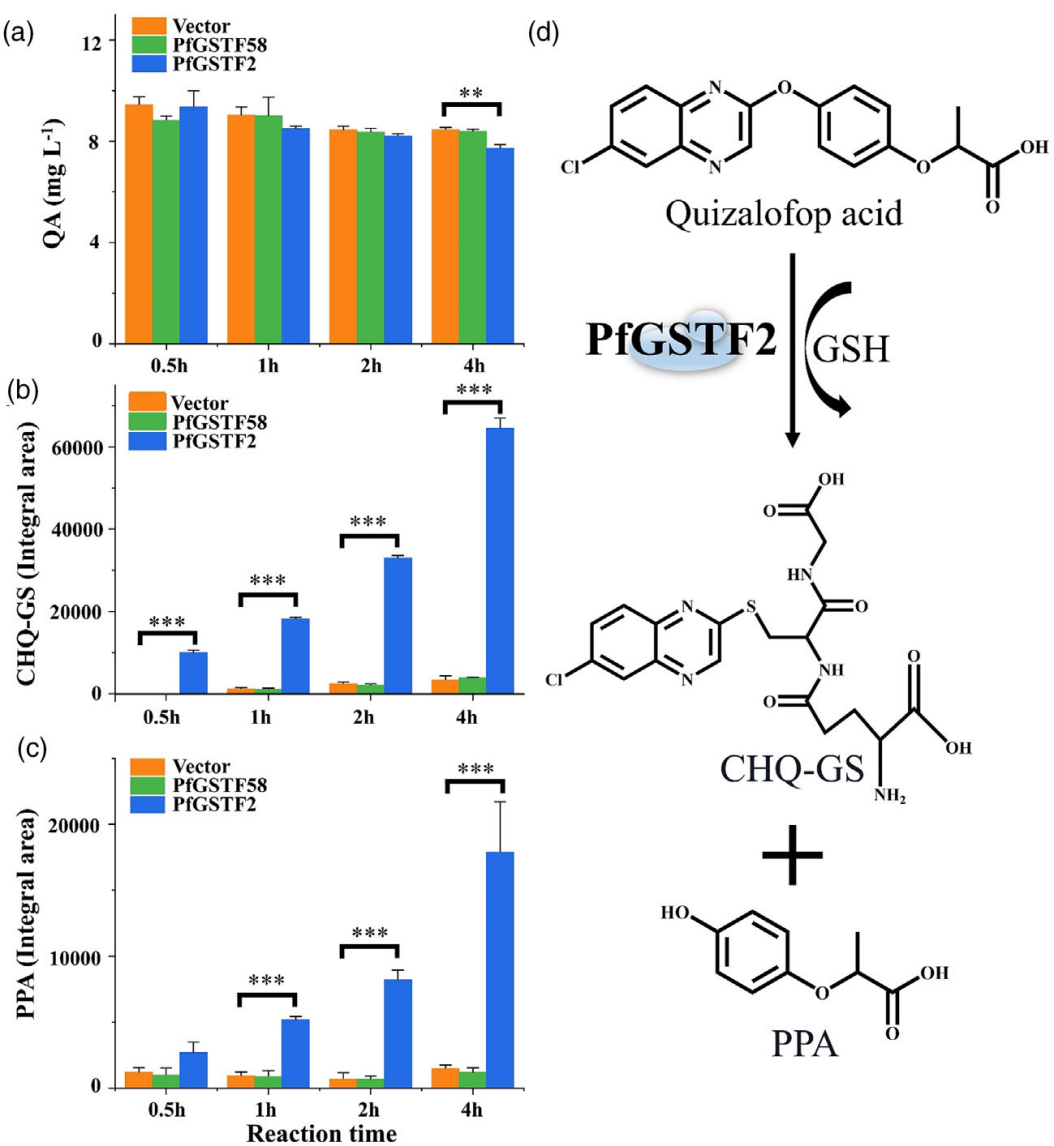
Time‐dependent *in vitro* metabolism of quizalofop acid by *E. coli*‐expressed PfGSTF2 or PfGSTF58 analysed by UHPLC‐Q‐TOF‐MS, 0.5, 1, 2 and 4 h after incubation with glutathione. (a) quizalofop acid (QA) remaining in the incubation mixture. (b) Metabolite 6‐chloroquinoxalin‐2‐ol‐glutathione (CHQ‐GS) conjugate. (c) Metabolite 2‐(4‐hydroxyphenoxy) propanoic acid (PPA). Data are means ± SE (*n* = 4). ** and *** indicate significant difference (*P* < 0.01 and 0.001, respectively) between vector control and PfGSTF2, by Student's *t*‐test. (d) Suggested detoxification pathway of quizalofop acid catalysed by PfGSTF2. Vector: Mixture of pET28a, glutathione and quizalofop acid.

**Figure 4:**
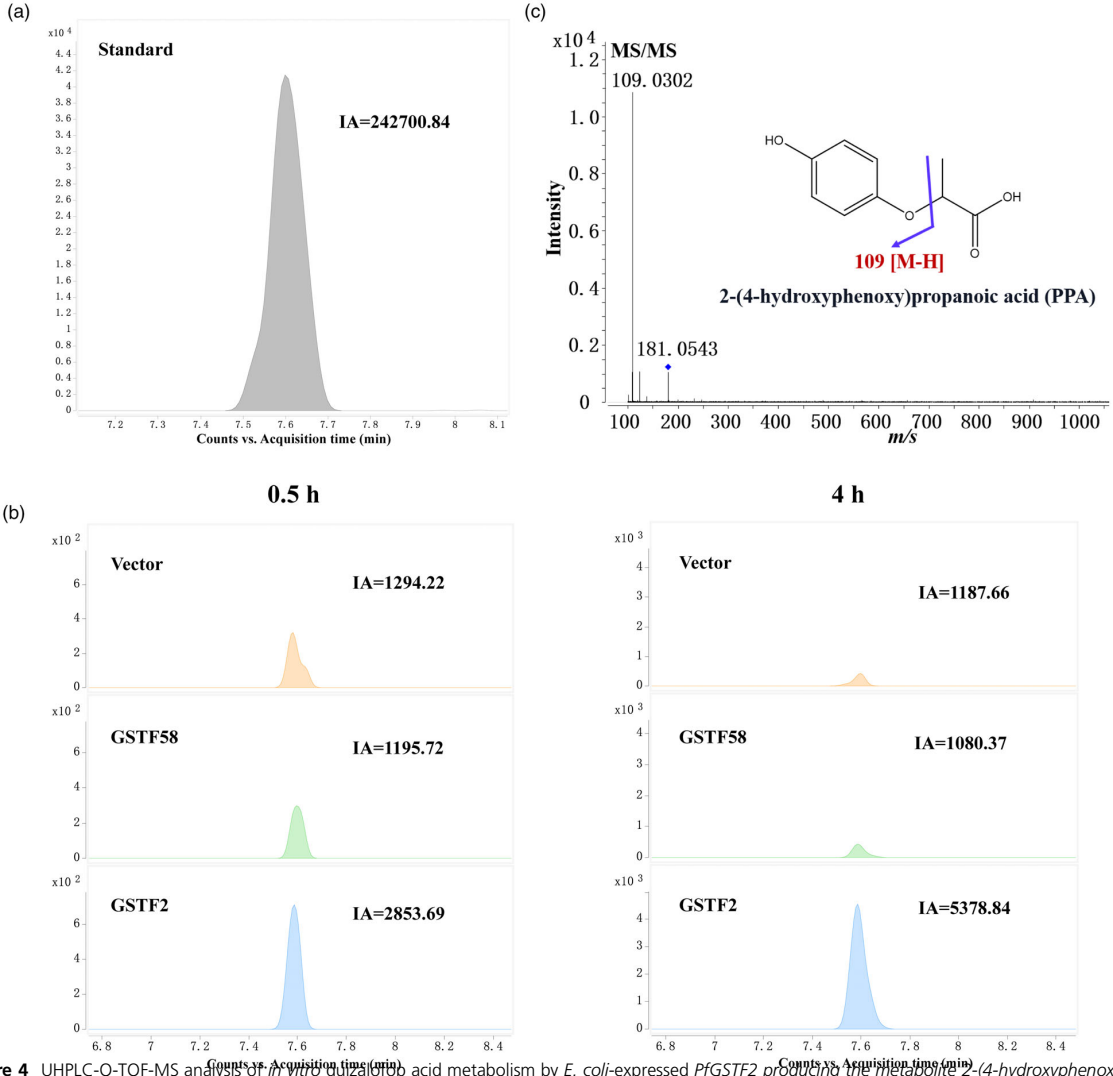
UHPLC‐Q‐TOF‐MS analysis of *in vitro* quizalofop acid metabolism by *E. coli*‐expressed PfGSTF2 producing the metabolite 2‐(4‐hydroxyphenoxy) propanoic acid (PPA). (a) Standard analytical grade PPA. (b) Quantification of PPA produced by *E. coli*‐expressed PfGSTF58 and PfGSTF2 in comparison to the vector pET28a, 0.5 and 4 h after incubation with glutathione and quizalofop acid. (c) Second‐order mass spectrum of PPA (peak at *m/z* 181.054). Vector: Mixture of pET28a, glutathione and quizalofop acid as a control. IA: integral area.

**Figure 5:**
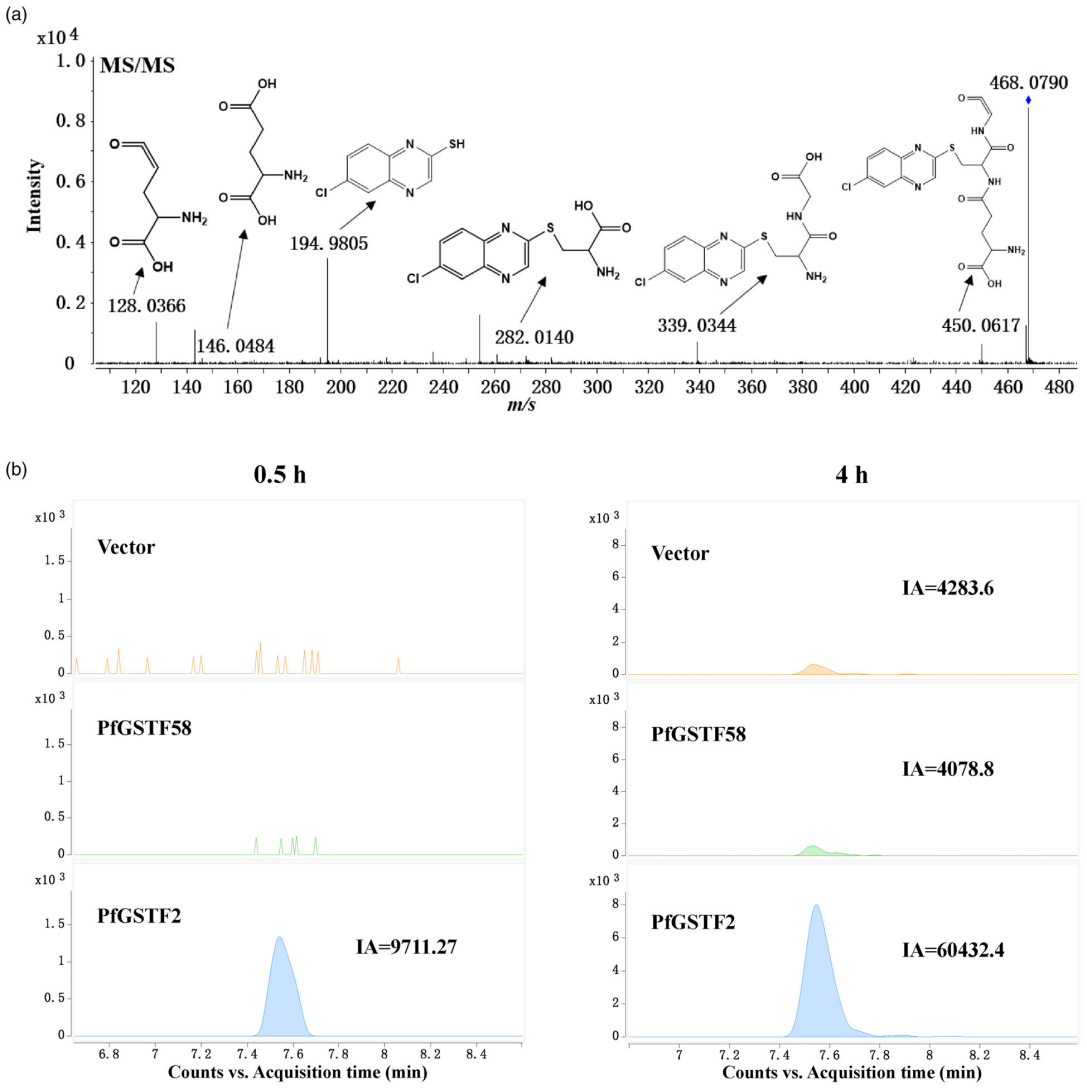
UHPLC‐Q‐TOF‐MS analysis of *in vitro* quizalofop acid metabolism by *E. coli*‐expressed PfGSTF2 producing the GST conjugation metabolite. (a) Second‐order mass spectrum of the quizalofop acid metabolite 6‐chloroquinoxalin‐2‐ol‐glutathione conjugate (CHQ‐GS, peak at *m/z* 468.079). Other peaks are fragmentation of the CHQ‐GS for analysis. (b) Relative levels of the metabolite CHQ‐GS produced by *E. coli*‐expressed PfGSTF2 or PFGSTF58, 0.5 and 4 h after incubation with glutathione and quizalofop acid. Vector: Mixture of pET28a, glutathione and quizalofop acid as a control. IA: integral area.

High‐resolution mass spectrometry was used to identify quizalofop acid metabolism and its potential metabolites by PfGSTF2. Quizalofop acid standard was identified by molecular ion peak at *m/z* 343.05, which was also verified by the fragment ion peaks (Figure S9). Compared to vector control and PfGSTF58, the quizalofop acid content was lower in the PfGSTF2 reaction system, especially 4 h after incubation (Figure 3a). Based on the second‐order mass spectrum full scan data, two possible metabolites were identified: 2‐(4‐hydroxyphenoxy) propanoic acid (PPA) (*m/z* 181.05) and 6‐chloroquinoxalin‐2‐ol‐glutathione (CHQ‐GS) (*m/z* 468.08) (Figures 4 and 5). CHQ‐GS is quizalofop acid, and GSH conjugate and PPA is a reaction derivative. The nature of PPA was further confirmed by comparing to an analytical standard (Figure 4). CHQ‐GS and PPA levels increased with incubation time in the PfGSTF2 reaction system and were significantly higher than those in the vector control and PfGSTF58 (Figure 3b, c). Trace amounts of CHQ‐GS and PPA in the vector control and PfGSTF58 were also detected, likely due to non‐enzymatic spontaneous conjugation of GSH to quizalofop acid (Bakkali *et al*., 2007).

In addition, there was no difference in quizalofop acid levels in any of the reaction groups in the absence of GSH, 8 h after incubation (Figure S10a), and the metabolites CHQ‐GS and PPA were not detected. This indicates that quizalofop acid *in vitro* metabolism by PfGSTF2 is enzymatic and GSH‐dependent. Therefore, a detoxification pathway for quizalofop acid catalysed by PfGSTF2 was proposed (Figure 3d). However, there was no *in vitro* metabolism of haloxyfop acid by PfGSTF2 and PfGSTF58 with no detectable amount of haloxyfop acid metabolites (Figure S10b).

### Analysis of quizalofop acid metabolite in plants of SC‐R and SC‐S

**Figure 6:**
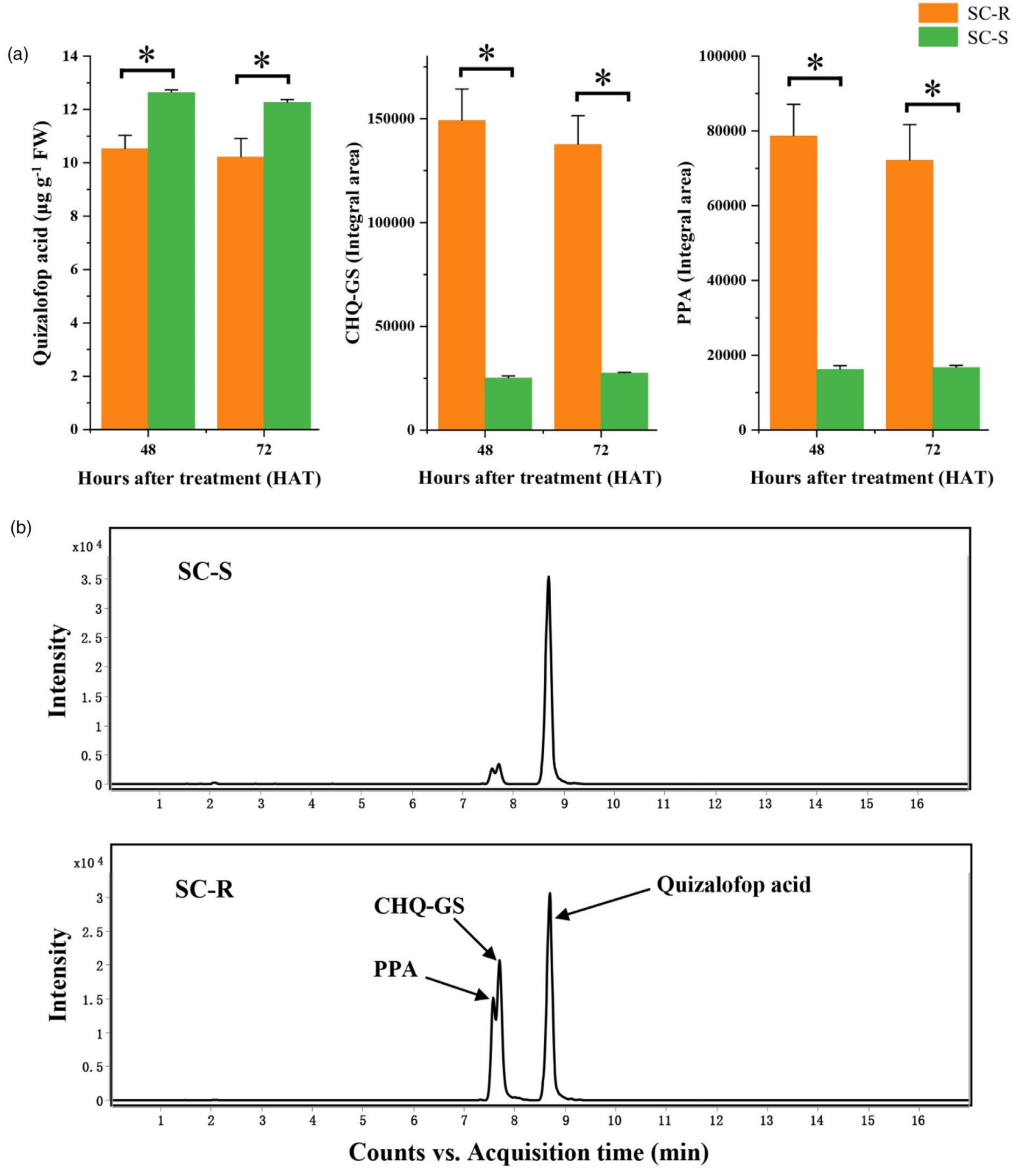
UHPLC‐Q‐TOF‐MS analysis of quizalofop acid metabolism in quizalofop‐p‐ethyl‐resistant (SC‐R) and quizalofop‐p‐ethyl‐susceptible (SC‐S) plants of *P. fugax*. (a) Levels of quizalofop acid, metabolites 6‐chloroquinoxalin‐2‐ol‐glutathione (CHQ‐GS) conjugate and 2‐(4‐hydroxyphenoxy) propanoic acid (PPA) in SC‐R and SC‐S *P. fugax* plants after treatment. (b) Representative scans of quizalofop acid, CHQ‐GS and PPA in SC‐R and SC‐S plants on LC–MS/MS, 48 h after treatment. Data are means ± SE of three biological replicates per time point. * indicates significant difference (*P* < 0.05) between SC‐R and SC‐S *P. fugax* plants, by Student's *t*‐test.

Quizalofop acid metabolism by PfGSTF2 and PfGSTF58 was analysed using liquid chromatography‐mass spectrometry (LC‐MS). The two quizalofop acid metabolites (CHQ‐GS and PPA) identified *in vitro* by PfGSTF2 (Figure 3b, c) were also detected in SC‐R and SC‐S plants. The levels of CHQ‐GS were up to 5.9 times (*P* < 0.05) higher, and the levels of PPA were up to 4.9 times (*P* < 0.05) higher in the SC‐R than the SC‐S plants, 48 and 72 h after treatment (Figure 6). In addition, as expected and determined before using LC–MS analysis (Figure 1a), quizalofop acid residue was significantly lower in SC‐R than in SC‐S plants (*P* < 0.05) (Figure 6a). These results are consistent with the *in vitro* results by PfGSTF2 (Figure 3), confirming quizalofop acid metabolism by PfGSTF2‐mediated herbicide conjugation.

### Prediction of PfGSTs structures and molecular docking

The predicted PfGSTF2 enzyme encompasses a GSH binding site (G‐site), responsible for binding the GSH cofactor, and an adjacent electrophilic binding site (H‐site), interacting with the electrophilic substrate (Figure S11a, b). GSH is stabilized through a three‐dimensional hydrogen bond network involving residues Gln54, Ile55, Glu67, and Ser68, and it also forms a salt bridge with Lys42 (Figure S11c). Like other plant Ser‐GSTs (Pegeot *et al*., 2014; Schwarz *et al*., 2021; Sylvestre‐Gonon *et al*., 2019), the conserved catalytically active residue Ser12 anchors the sulfhydryl group of GSH (Figure S12a, b), playing a crucial role in GSH conjugation activation (Cummins *et al*., 2011). Notably, the conformation of the active pocket at the H‐site varies between PfGSTF58 and PfGSTF2. During the docking of the known substrate CDNB, the optimal binding conformation of PfGSTF58 exhibits a distance of 3.3 Å between the sulfhydryl group of GSH and the Cl atom of CDNB, smaller than that in PfGSTF2 (5.9 Å) (Figure S12). This indicates that substitution reactions are more susceptible in PfGSTF58, possibly explaining its higher affinity for CDNB than PfGSTF2 (Figure S7).

For the docking of the herbicide quizalofop acid in PfGSTF2, hydrophobic interactions form with Phe122, Met126, Arg127 and Tyr175, and hydrogen‐bonding interactions occur with Thr13, Asn14 and Tyr175 (Figure S11d), resulting in a binding energy of −6.50 kcal mol^−1^ less than that of quizalofop acid with PfGSTF58 (−5.75 kcal mol^−1^). Docking results suggest that quizalofop acid binds more firmly in the active pocket of PfGSTF2, possibly facilitating the coupling of GSH to quizalofop acid. Moreover, the smaller distance (<5 Å) between the sulfhydryl group of GSH and O‐atoms of herbicides in PfGSTF2 makes the sulfhydryl group of GSH easier to exert nucleophilic attack on the electrophilic centres of quizalofop acid, compared to PfGSTF58 (Figure S12c, d). This difference in binding and reactivity between PfGSTF2 and PfGSTF58 contributes to their distinct enzymatic activities and substrate specificities.

### Reduced herbicidal effect of 2‐(4‐hydroxyphenoxy) propanoic acid (PPA)

**Figure 7:**
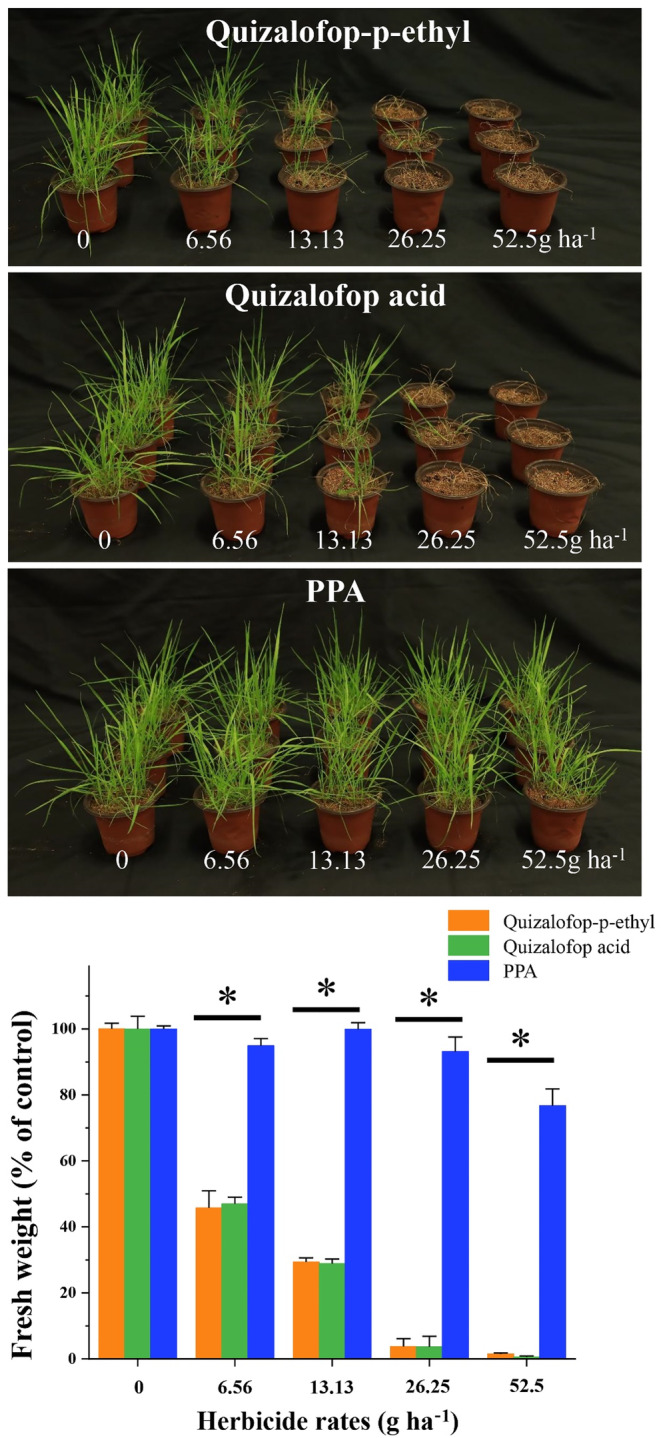
Reduced herbicidal activity of the quizalofop metabolite 2‐(4‐hydroxyphenoxy) propanoic acid (PPA) as compared to quizalofop‐p‐ethyl and quizalofop acid. Seedlings quizalofop‐p‐ethyl‐susceptible (SC‐S) plants of *Polypogon fugax* were treated with quizalofop‐p‐ethyl, quizalofop acid and 2‐(4‐hydroxyphenoxy) propanoic acid (PPA), respectively, at the 3‐ to 4‐leaf stage. The experiment was conducted with three replicates (ten plants per replicate) per treatment. Photos were taken 3 weeks after treatment. Data are means ± SE of three biological replicates per treatment. * indicates significant difference (*P* < 0.05) by Student's *t*‐test.

As the GSH conjugate (CHQ‐GS) of quizalofop acid is expected to have reduced herbicidal activity, we only tested the herbicide activity of the metabolite PPA in *P. fugax* seedlings. As shown in Figure 7, PPA caused much less growth reduction (shoot fresh weight) across the rates used (ranging from 5.1 ± 2.2 to 23.3 ± 5.1%), as compared to quizalofop‐p‐ethyl (ranging from 54.3 ± 5.2 to 98.5 ± 0.3%) and quizalofop acid (ranging from 53.0 ± 2.1 to 99.4 ± 0.4%). It is evident that metabolite PPA has much less herbicidal activity than the parent herbicide.

### PfGSTF2 and PfGSTF58 could relieve the oxidase stress in *Escherichia coli* and rice calli

To reveal if PfGSTF2 and PfGSTF58 have a role in antioxidant stress, the disc diffusion assay for reactive oxygen species (ROS) damage was performed with transformed *Escherichia coli* culture. The halo diameter of the inhibition zone around the filter disc was positively correlated with CHP concentrations on pET‐28a (+) (vector alone) or pET‐28a (+)/PfGSTF transformed LB plates (Figure S13a). The inhibition zones of pET‐28a (+)/PfGSTF58 or pET‐28a (+)/ PfGSTF2 were significantly smaller than those of pET‐28a (+) transformed LB plates (Figure S13a). At various CHP concentrations, the halo diameters were reduced by up to 44% for pET‐28a (+)/ PfGSTF58 and up to 33% for pET‐28a (+)/ PfGSTF2, respectively, compared to pET‐28a (+). In addition, similar GSH‐dependent peroxidase (GPOX) activities were observed for PfGSTF58 and PfGSTF2 (*P* > 0.05) (Figure S13b).

In transgenic rice calli, there was no significant difference in the malondialdehyde (MDA) content in any callus lines without quizalofop‐p‐ethyl treatment. However, at 10 nM treatment, MDA content was significantly increased in all three lines, with the highest increase in the *GFP*‐OE line (Figure S13c). In addition, ROS production (hydrogen peroxide) in transgenic rice calli after quizalofop‐p‐ethyl (10 nM) was examined by DAB staining. There was no visual difference in ROS staining in all lines without herbicide treatment (Figure S13d). However, under 10 nM treatment, both *PfGSTF58*‐OE and *PfGSTF2*‐OE lines had lighter DAB staining than the *GFP*‐OE lines (Figure S13d). These results indicate that quizalofop‐p‐ethyl treatment did cause oxidative stress in *E. coli* cells and rice calli, and *PfGSTF58* and *PfGSTF2*‐overexpressing cells and calli did suffer less damage.

### Antioxidant capacity in SC‐R and SC‐S populations

DAB staining was slightly lighter in the SC‐R than in the SC‐S plants in the control without herbicide treatment (Figure S14a). At 3 days after herbicide treatment, DAB staining was much darker in the SC‐S than in the SC‐R plants (Figure S14a). In addition, MDA content increased significantly in the SC‐S plants (Figure S14b). Basal levels of GPOX activity were significantly, but not remarkably, higher in SC‐R than SC‐S plants (Figure S14c). However, 3 days after herbicide treatment, the GPOX activity of SC‐R and SC‐S plants was the same (Figure S14c). These results suggest that quizalofop‐p‐ethyl treatment did cause plant oxidative stress by producing ROS, and attenuation of such stress may also play a role, if not much, in SC‐R resistance to the herbicide.

## Discussion

Previous research on the *AmGSTF1* gene in *A. myosuroides* demonstrated its involvement in multiple herbicide resistance to chloroxuron, metolachlor and atrazine (Cummins *et al*., 2013). *AmGSTF1* orthologues in *L. rigidum* (Torra *et al*., 2021) and *Beckmannia syzigachne* (Bai *et al*., 2020), and *GSTF11 in Leptochloa chinensis* (L.) Nees (Zhang *et al*., 2022) have been recently implicated in herbicide resistance (particularly ACCase‐inhibiting herbicides). In addition, in waterhemp, *AtuGSTF2* may be related to the metabolic resistance to atrazine (Evans Jr. *et al*., 2017). More recently, it has been reported that two phi‐class GSTs detoxify flufenacet via the formation of flufenacet‐glutathione conjugate *in vitro* and have a similar GST activity against fenoxaprop‐p‐ethyl (Parcharidou *et al*., 2023). Thus, the research on the phi‐class GSTs in herbicide resistance has been increasingly.

In the current study, two phi‐class GST genes (*PfGSTF2* and *PfGSTF58*) were identified in an herbicide‐resistant *P. fugax* population, which share amino acid identity of over 85%. Despite this high sequence similarity, the two GSTs displayed contrasting response to the studied herbicides in transgenic rice calli. The resistance pattern of *PfGSTF2* transgenic rice calli is consistent with the *P. fugax* SC‐R population (Chen *et al*., 2020). Furthermore, the activity of PfGSTF2, but not PfGSTF58, was significantly inhibited (6‐fold) (*P* < 0.05) by the GST inhibitor NBD‐Cl at higher concentrations (Figure S8), and this explains why NBD‐Cl reversed the quizalofop‐p‐ethyl resistance in the SC‐R population in our previous study (Chen *et al*., 2020). All these results suggest that PfGSTF2 is the main player for the herbicide resistance in the SC‐R *P. fugax* population. Importantly, CRISPR/Cas9 knockout of the rice OsGSTF2 (orthologue of PfGSTF2) significantly (*P* < 0.05) increased rice seedling sensitivity to quizalofop‐p‐ethyl, highlighting the functional conservation of this GST in grasses (Figure S6), and further validating the role of PfGSTF2 in herbicide resistance. This may provide an opportunity for genetic manipulation of the GST gene to achieve herbicide tolerance in crop species.

While overexpression of the *PfGSTF2* gene gives herbicide tolerant phenotypes in transgenic rice calli and seedlings (Figures 2 and S4), what is the underling metabolic base of this resistance? The *in vitro* metabolic activity assay revealed that PfGSTF2 had significant conjugating activity towards quizalofop acid, distinguishing it from PfGSTF58 which has higher activity against the standard substrate CDNB (Figures 3a and S7). This indicates that GST activity against CDNB and the herbicide metabolism are not consistently correlated. Indeed, in a study examining GST activity in different tissue of annual ryegrass seedlings, there was no correlation between GST activity against CDNB and that against pyroxasulfone in pyroxasulfone‐resistant and pyroxasulfone‐susceptible populations (Goggin *et al*., 2021). Therefore, GST activity against CDNB as a criterion for determining GST involvement in herbicide resistance needs to be interpreted carefully (Cummins *et al*., 2009; Wang *et al*., 2020, 2021).

The Phi‐class GSTs are known for their roles in detoxifying exogenous compounds by facilitating the GSH conjugation of electrophilic molecules. Indeed, our MS analysis of *in vitro* quizalofop acid metabolism by recombinant PfGSTF2 revealed a positive correlation between the level of CHQ‐GS conjugate and the presence of PPA over reaction time (Pearson's *r* = 0.95, *P* < 0.001) (Figure 3). This suggests that recombinant PfGSTF2 catalyses CHQ‐GS conjugation on the ether bond of the quizalofop acid, leading to generation of PPA. Conversely, while trace amount of CHQ‐GS and PPA was detected in recombinant PfGSTF58 at the baseline level due to non‐enzymatic reactions, similar to that in the vector control reaction group (Figure 3). This confirms the lack of capacity of PfGSTF58 in metabolizing quizalofop acid. Prior investigations into fenoxaprop‐p‐ethyl metabolism indicated that all recombinant GST enzymes facilitate GSH binding either directly via fenoxaprop‐p‐ethyl or via the chlorobenzoxazolone (CDHB, structures conjugated with GSH) intermediate (Parcharidou *et al*., 2023). However, the origins of the CDHB intermediate (whether arising from spontaneous or enzymatic degradation of fenoxaprop‐p‐ethyl or uncoupling of CDHB‐glutathione conjugates) remain unclear. In our experiments, the CHQ intermediate was not detected in any treatment groups with and without GSH, indicating that GSH reacts enzymatically and directly on the substrate quizalofop acid to form the stable conjugate CHQ‐GS (Figure 3d). Equally, a higher level of CHQ‐GS and PPA along with a lower level of quizalofop acid residue was detected in the SC‐R *P. fugax* population relative to the SC‐S population after herbicide treatment (Figure 6). Furthermore, we found the quizalofop acid metabolite PPA had lower *in vivo* herbicidal activity (Figure 7), while the quizalofop acid metabolite CHQ‐GS is expected to have lower herbicidal activity than PPA due to GSH conjugation. Therefore, all these experimental evidences confirm that overexpression of *PfGSTF2* resulted in enhanced metabolism of quizalofop acid to the less or non‐toxic GSH conjugate and derivative, thereby endowing plants with quizalofop‐p‐ethyl resistance.

It is known that the biotransformation of xenobiotics via GSH conjugation constitutes is a Phase II detoxification mechanism and may depend on Phase I activation (Concepcion *et al*., 2021; Edwards *et al*., 2000; Mano *et al*., 2019; Theodoulou *et al*., 2003). For instance, it was found that conjugated metabolites of isoproturon and acetochlor in rice underwent modification by Phase I enzymes (such as P450, methyltransferase or hydrolases family) before conjugation with glycose, amino acids or GSH (Su *et al*., 2023; Zhai *et al*., 2022). Work by Concepcion *et al*. (2021) in *A. tuberculatus* revealed syncarpic acid‐3 is sequentially catalysed by reductase and dehydratase enzymes, promoting the formation of GSH conjugates (SA3‐GSH) during Phase II detoxification. Similarly, Suda *et al*. (2023) reported the formation of glycosylated metabolites of diclofop‐methyl in *E. phyllopogon* relies on pre‐hydroxylation by specific P450 enzymes. Nevertheless, in our study, *in vivo* and *in vitro* experiments indicated that quizalofop acid metabolism by PfGSTF2 is likely via direct GSH conjugation, independent of Phase I activation (Figures 3 and 6), akin to the GST‐mediated metabolism of flufenacet, fenoxaprop‐p‐ethyl, or pyroxasulfone (Goggin *et al*., 2021; Parcharidou *et al*., 2023). Moreover, overexpression of *PfGSTF2* also conferred resistance to haloxyfop‐R‐methyl (Figure 2b). Nonetheless, haloxyfop‐R‐methyl degradation and direct GSH conjugates were not detected *in vitro* by recombinant PfGSTF2 (Figure S10b). This suggests that PfGSTF2‐mediated resistance to haloxyfop‐R‐methyl may differ from that of quizalofop‐p‐ethyl, and could be dependent on Phase I enzyme activation and/or other factors.

Given the evidence that PfGSTF2 (but not PfGSTF58) can catalyse direct GSH conjugation of quizalofop‐p‐ethyl, then what is the structural basis of this differential reaction? By comparing the herbicides binding sites of PfGSTF2 and PfGSTF58, a larger closed three‐dimensional mesh formed by residues amino acid 33 to amino acid 41 in PfGSTF2 was better suited to bind quizalofop acid than PfGSTF58 (Figure S11b). This region exhibits multiple amino acid substitutions among comparing sequences (Figure S15), suggesting an important role in its spatial preference for substrate binding, although the contribution of other sites cannot be ruled out. Similarly, it has been indicated that the greater pesticide detoxification capacity of GSTe2 in insects may be attributed to a larger substrate binding pocket and a better hydrophobic seal (Low *et al*., 2010; Lu *et al*., 2023; Wang *et al*., 2008). The molecular docking experiment provides a preliminary explanation into the quizalofop acid metabolism by PfGSTF2 versus PfGSTF58. Future experiments such as crystal structure and targeted mutagenesis of PfGSTF2 can help identify key residues for substrate binding and catalysis to achieve higher enzyme activity.

In addition to GSH conjugation, GSTs also have antioxidant capacity against herbicide‐induced oxidative stress (Cummins *et al*., 2013; Powles and Yu, 2010). ROS are produced by both plant and animal cells as a part of normal cellular processes and in response to stress conditions (Zandalinas and Mittler, 2018). At low concentrations, ROS can act as signalling molecules, but at high concentrations, they can be toxic to cells. When plants are exposed to various stresses such as salinity, drought, heavy metals and herbicides, the level of ROS in the plant increases, leading to damage to cell membrane components, ultimately affecting plant growth (Cummins *et al*., 1999; Gill and Tuteja, 2010). Peroxidases oxidize various substrates utilizing hydrogen peroxide (H_2_O_2_) or organic hydroperoxides, and thus, they are involved in scavenging of ROS (Bela *et al*., 2015). GST not only mitigates phytotoxicity of exogenous toxic compounds through GSH conjugation, but also plays a role in antioxidant stress by possessing the GPOX activity. The antioxidant capacity of GSTs enzymes (as GPOX) is important in plant responses to adversities (Cummins *et al*., 2009; Gao *et al*., 2022; Roxas *et al*., 1997; Zhang *et al*., 2021) and even in insect responses to pesticides (Li *et al*., 2021). For instance, studies in *A. myosuroides* proposed that herbicide resistance is largely dependent on the ability of *AmGSTF1* to scavenge hydroperoxides released downstream of herbicide injury (Cummins *et al*., 2013). In our study, the basal level of GPOX activity of SC‐R population slightly but significantly (*P* < 0.05) higher than that of SC‐S (Figure S14c). Although this GPOX activity was induced in the SC‐S population by herbicide treatment, higher basal GPOX activity in the SC‐R might facilitate prompt response to herbicide‐induced ROS stress. Indeed, the *PfGSTF2* gene possesses the GPOX activity that protects *E. coli* against CHP and rice calli against herbicides (Figure S13). Interestingly, *PfGSTF58* also has similar antioxidant capacity as *PfGSTF2*, and this might contribution to weak resistance to quizalofop‐p‐ethyl of *PfGSTF58‐*expressing rice calli (Figure 2a).

In summary, the key findings of our study are the identification and characterization of a phi‐class GST enzyme (PfGSTF2) that endows resistance to the ACCase‐inhibiting herbicides by GSH conjugation and to a less extent, by antioxidant activity. Based on this, a mechanistic framework for herbicide resistance in resistant SC‐R *P. fugax* was proposed (Figure S16). Quizalofop‐p‐ethyl enters into *P. fugax* plant cells and is activated by non‐specific esterase to form the active quizalofop acid. Enhanced expression of the *PfGSTF2* gene elevates the production of PfGSTF2 enzyme in plants of SC‐R population. PfGSTF2 detoxifies quizalofop acid by GSH conjugation, lowering quizalofop acid to a sublethal level entering the ACCase target site. In addition, the slightly higher constitutive level of *PfGSTF2* GPOX activity may also have advantages scavenging herbicide‐induced ROS, mitigating cytotoxicity.

Our study advances the knowledge of GST‐mediated xenobiotic resistance in general, and NTSR‐based metabolic herbicide resistance, in particular, enzymes in herbicide metabolic resistance. In practice, as GSTs can have a broad spectrum of substrate specificity (i.e. can metabolize multiple herbicides), herbicide mixtures and rotations with ACCase‐inhibiting herbicides need to be careful to avoid GST‐mediated metabolism. As genome editing of the orthologue GST gene (*OsGSTF2*) can modify crop sensitivity to the studied herbicide, it also opens up possibilities for engineering herbicide tolerance in crops through genetic manipulation of GSTs. Further work is to determine how common this *PfGSTF2* is involved in herbicide resistance in other *P. fugax* populations, and investigate the regulatory network of *PfGSTF2* and its homologues in herbicide resistance and other abiotic stresses. Inclusion of multiple weed management strategies (such as non‐chemical control) needs to be considered to fight diverse herbicide resistance mechanisms in weed populations.

## Materials and methods

### Plant materials and growth conditions

The quizalofop‐p‐ethyl‐resistant (SC‐R) and quizalofop‐p‐ethyl‐susceptible (SC‐S) populations of *P. fugax* were original from Sichuan Province in China (Chen *et al*., 2020). The resistance of the SC‐R population did not involve mutations in the ACCase gene but exhibited GST‐based NTSR (Chen *et al*., 2020). Seed germination, growth conditions and herbicide treatments were detailed in the previous study (Chen *et al*., 2020).

### UHPLC analysis of quizalofop acid levels in *P. fugax* populations

Quizalofop‐p‐ethyl is rapidly converted to the active form quizalofop acid by ester hydrolysis once it enters in plants (Lopez‐Ruiz *et al*., 2017, 2018). Quizalofop acid content was determined by UHPLC in plants of SC‐R and SC‐S populations. At the 4–5 leaf stage, 20 μL treatment solution (dilution of technical grade quizalofop‐p‐ethyl in methanol) at a concentration of 50 mg L^−1^ (corresponding to 16 g ha^−1^) was applied to two leaves of each plant by micro‐pipetting. Above‐ground plant tissue was collected at 1, 3, 5, 7 and 9 d after treatment, and 10 plants were pooled as one sample and there were 3 samples per time point per population. The samples were harvested, rinsed in water and grounded in liquid nitrogen with steel beads (see our previous studies for details of sample pre‐treatment) (Mo *et al*., 2022), and quizalofop acid content was quantified using UHPLC. The chromatographic separation was carried out as described (Liu *et al*., 2020), with a 20 μL aliquot injection. The mobile phase consisted of 70% methyl alcohol and 30% ultrapure water (v/v) containing 0.1% acetic acid. Each time point involved 3 replicate samples, and the experiment was carried out twice. The datasets from repeated experiments were analysed by SPSS v23 (IBM, Armonk, USA) using the general linear model procedure for variance analysis. There was no significant difference between repeated experiments (*P* > 0.05). Technical and analytical grade quizalofop acid was used for quantification.

### Full‐sequence cloning and analysis of the candidate GST genes

Constitutive overexpression of the two GST genes (*GST2c* and *GSTL3*) was associated with herbicide resistance in SC‐R *P. fugax* in our previous study (Chen *et al*., 2020). Total RNA was extracted from the leaf tissue of the SC‐R plants using RNAiso Plus (TaKaRa Biotech, China). The first‐strand cDNA synthesis and amplification procedure were performed as previously described, using the HiScript II First Strand cDNA Synthesis Kit (Vazyme biotech, USA) and 2× PrimeSTAR Max Premix (TaKaRa, Japan), respectively. Given the higher expression of *GST2c* than *GSTL3*, we focused our subsequent investigations on *GST2c*. Due to lack of genomic information for *P. fugax*, we employed a homologous cloning strategy based on the known partial sequences (HF548530.1, KM062112 and MN936108.1 in the GenBank database) to design specific primer pairs (Table S1) for the full‐length amplification of *GST2c*. The *GST2c* genes of the SC‐R and SC‐S populations were cloned and sequenced as described previously (Chen *et al*., 2023). A phylogenetic tree was constructed with 1000 bootstrap replicates using the MEGA 5.0 software (MEGA Inc., Englewood, NJ) between *P. fugax GST2c* genes and the wheat genome (Wang *et al*., 2019). The conserved motifs in the *P. fugax* and wheat GSTs were predicted by the MEME program (http://meme‐suite.org/tools/meme) (Bailey *et al*., 2009), with the maximum number of motifs set at 10 and the width ranges from 15 to 50.

### Expression levels of *GSTF2* and *GSTF58* genes in *P. fugax* populations

Expression levels of *PfGSTF2* and *PfGSTF58* were determined in SC‐R and SC‐S *P. fugax* populations to test whether both variants are involved in herbicide resistance. When SC‐R and SC‐S *P. fugax* seedlings reached the tillering stage, leaf tissues were collected at 0 and 72 h after quizalofop‐p‐ethyl treatment (at 6.56 g a.i. ha^−1^), a non‐lethal dose for *P. fugax* (Chen *et al*., 2023). The herbicide was applied using a 3WP‐2000 mobile sprayer equipped with a 390 mL min^−1^ flow nozzle TP6501E delivering 372 L ha^−1^ at a pressure of 3.0 kg cm^−2^. Total RNA was extracted as described above and specific primer pairs (Table S1) were designed to distinguish these two variants. Total RNA (2 μg) of *P. fugax* was reverse transcribed using the HIScript II Q RT SuperMix (Vazyme, Nanjing, China) for the quantitative real‐time PCR (RT‐qPCR). The relative expression levels of the *PfGSTF2* and *PfGSTF58* between the SC‐R and SC‐S populations were calculated by the 2^−ΔCt^ method, using the previous reported *elongation factor‐1 alpha* (*EF‐1α*) as an internal reference gene (Chen *et al*., 2020).

### Rice callus transformation and growth response to the herbicides

The expression cassettes of *PfGSTF2* and *PfGSTF58* were generated following the previously established method (Pan *et al*., 2019). Briefly, *PfGSTF2*, *PfGSTF58* or *GFP* was incorporated into the transformation vector POX, driven by the 35S promoter, generating the *PfGSTF* overexpression (*PfGSTF2*‐OE, *PfGSTF58*‐OE) and *GFP* overexpression (*GFP*‐OE) vectors. Prior to rice transformation, the constructed vectors were checked for correct insertion by DNA sequencing. Rice callus transformation was conducted as described (Toki *et al*., 2006), using hygromycin (50 mg L^−1^) for selection. The hygromycin phosphotransferase (HPT) gene in the vector was amplified to confirm the introduction of the target gene into the rice calli, as previously described (Pan *et al*., 2019). Ten hygromycin‐resistant rice calli were transferred to fresh N6D solid media supplemented with the 4 ACCase inhibitor herbicides (quizalofop‐p‐ethyl, 0–80 nM, fenoxaprop‐p‐ethyl, 0–4 μM, pinoxaden, 0–160 nM or haloxyfop‐R‐methyl, 0–400 nM). Growth response to herbicides of *PfGSTF2*‐OE, *PfGSTF58*‐OE and *GFP*‐OE calli was compared 3 weeks after treatment and the experiment was carried out twice.

### Quizalofop‐p‐ethyl sensitivity of transgenic rice seedlings

Transgenic rice calli (*GFP* and *PfGSTF2* overexpressing lines) were regenerated, and T_0_ plantlets ranging from 3 to 5 cm in length were transferred to rooting medium supplemented with hygromycin. After 7 days of acclimatization, the T_0_ seedlings were transferred to a mixture of fertilized soil and perlite (2:1, v/v) for subsequent molecular analysis and herbicide resistance testing as previously described (Pan *et al*., 2019). T_0_ seedlings of 18 plants (nine plants per treatment) were then foliar‐treated with quizalofop‐p‐ethyl at 0 and 26.3 g ha^−1^ using a 3WP‐2000 mobile sprayer as described in Section ‘Expression levels of *GSTF2* and *GSTF58* genes in *P. fugax* populations’. Plant shoot fresh weight was determined three weeks after treatment.

### Rice *OsGSTF2* gene knockout by CRISPR/Cas9 gene editing

A 23‐bp targeting sequence was selected, and the rice genome (http://blast.ncbi.nlm.nih.gov/Blast.cgi) was used for blast search to confirm targeting specificity (Hsu *et al*., 2013), and then, the construct was integrated into the pBGK032 vector. CRISPR/Cas9 plasmid introduction and rice transformation were performed as described (Toki *et al*., 2006). PCR amplification was performed using DNA extracted from the transformants and primers flanking the target site. The *OsGSTF2* gene was sequenced in all T1 transgenic lines and homozygous mutants were identified to generate T2 *OsGSTF2* KO lines (*osgstf2*). Herbicide susceptibility assays were performed on *osgstf2* and WT plants in test tubes as described previously (Maeda *et al*., 2019). Briefly, germinating rice seedlings were grown for 7 days in the half strength MS medium containing quizalofop‐p‐ethyl at 0, 20 and 40 nM, and shoot elongation of *osgstf2* and WT seedlings was compared. Six rice seedlings were used for each treatment, and the experiment was repeated.

### Heterologous expression of *PfGSTF2* and *PfGSTF58* in *E. Coli*

The full coding sequences of *PfGSTF2* and *PfGSTF58* were amplified with specific primers (Table S1), and the genes were cloned into the pET‐28a (+) vector, and the constructs were introduced into the *E. coli* BL21 (DE3) strain (Tsingke, Beijing, China) (Figure S17). Expression of PfGSTF2 and PfGSTF58 was induced by published procedures (Cho and Kong, 2005). The *E. coli*‐expressed crude enzymes were purified using a His‐tag protein purification kit (Coolaber, Beijing, China), at a concentration gradient of 0–500 mM imidazole. The purified protein was concentrated through 10 kDa molecular weight ultrafiltration tubes (Coolaber, Beijing, China) for 40 min at 4 °C. The protein was diluted in 50 mM PBS (pH 7.0) and quantified using the BCA protein assay kit (Coolaber, Beijing, China) and visualized by denaturing SDS‐PAGE.

### Enzyme kinetics of the recombinant protein PfGSTF2 and PfGSTF58

Kinetic parameters (*Km* and *Vmax*) for the standard substrate CDNB were measured in a 200 μL reaction with recombinant proteins, varying CDNB concentrations, and GSH. Absorbance at 340 nm was monitored at 30 °C, and the kinetic data were calculated using the Michaelis–Menten equation (SigmaPlot 10.0, Systat Software, Inc., San Jose, CA, USA). The effect of the GST inhibitor NBD‐Cl on enzyme activity was also assessed using the same setup, with NBD‐Cl replacing CDNB (Yang *et al*., 2009). Three replicates were included for each NBD‐Cl concentration.

### *In vitro* herbicide metabolism by recombinant PfGSTF2 and PfGSTF58

The reaction system consisted of 4 mM fresh GSH, 5 μg purified PfGSTF2 or PfGSTF58 and 30 μM quizalofop acid or haloxyfop acid in 50 mM PBS (pH 7.0). To identify quizalofop acid metabolites by PfGSTF2 and PfGSTF58, reactions were incubated at 37 °C and then terminated at different times (0.5, 1, 2 and 4 h) by adding an equal volume of acetonitrile. The solution was mixed at 200 rpm (Thermostatic shaker, Yiheng, China) for 30 min and the supernatant was obtained by centrifugation at 10 844 × *g* for 10 min, filtered through 0.22‐μm filter paper and used for the LC–MS analysis. Each reaction had four technical replicates. In addition, the treatment group without GSH was used as a control.

LC–MS analysis was conducted using the Agilent 1290 LC system coupled with a 6530 Q‐TOF/MS accurate‐mass spectrometer (Agilent Technologies, USA). A 4 μL sample was separated on a C18 column using a gradient of 0.1% formic acid aqueous solution (A) and acetonitrile (B) over 17 min. The flow rate was 0.3 mL min‐1, and the column was maintained at 20 °C. Electrospray ionization in both positive and negative modes allowed detection of molecular and fragment ions, confirming the identity of quizalofop acid and its metabolites. Mass accuracy was kept within 5 ppm, and compounds were quantified by their peak areas.

### Analysis of quizalofop acid metabolites in plants of SC‐R and SC‐S populations

At the 4–5 leaf stage, 20 μL of treatment solution (dilution of technical grade quizalofop acid in methanol) at a concentration of 50 mg L^−1^ (corresponding to 16 g ha^−1^) was applied by micro‐pipetting to two leaves of each plant. Above‐ground plant tissues were collected at 48 and 72 h after treatment, and the shoot material of 10 plants (about 400 mg fresh weights) was pooled as one sample and there were three samples per time point per population. Tissue quizalofop acid and its metabolites were extracted based on the published method (Suda *et al*., 2023) with modifications. The harvested samples were rinsed in water and grounded in liquid nitrogen with steel beads, then extracted with 5 mL of 80% cold methanol (v/v), followed by centrifugation at 10 000 × *g* for 10 min at 4 °C. The pellet was further extracted twice with 2 mL of 80% cold methanol (v/v), and the supernatant was pooled and evaporated under nitrogen at 40 °C for 30 min to remove methanol. The remaining aqueous phase was purified by an SPE‐C_18_ column (Lumeng, Jiangsu, China) and washed with 4 mL of water containing 0.1% formic acid (v/v), followed by elution with 4 mL of acetonitrile. The collected eluent was passed through a 0.22‐μm filter (Biosharp, Guangzhou, China) for high‐resolution LC‐Q‐TOF‐MS/MS detection, which was described above in section ‘*In vitro* herbicide metabolism by recombinant PfGSTF2 and PfGSTF58’. The experiment was repeated.

### Structural modelling and molecular docking of PfGSTF2 and PfGSTF58

The structural models of PfGSTF2 and PfGSTF58 were predicted using the swiss‐model server (http://swissmodel.expasy.org/). The crystal structure of *A. myosuroides* GSTF, complexed with the GSH cofactor (PDB code: 7odm), was selected as the template due to its high sequence identity with PfGSTF2 (85.4%) and PfGSTF58 (87.2%). The 3D structures of PfGSTF2 and PfGSTF58 were constructed, yielding global model quality estimation (GMQE) scores of 0.92 and 0.93, respectively, with a 90.5% and 93.9% of residues residing in favourable regions as assessed by the Ramachandran plot, indicating a high‐quality protein model. Molecular docking of PfGSTF2 and PfGSTF58 with CDNB and quizalofop acid was executed using AutoDock Tools (version 1.5.6), applying a flexible ligand and rigid receptor model in accordance with our established protocol (Chen *et al*., 2023). A Lamarckian genetic algorithm generated twenty independent conformations, and PyMOL (version 2.3.0) facilitated the visualization of the poses exhibiting the highest affinity scores. The interaction of PfGSTF2 and PfGSTF58 with the ligands was characterized using the Protein–Ligand Interaction Profiler (PLIP) server (Adasme *et al*., 2021).

### Herbicidal effect of the quizalofop acid metabolite in *P. fugax* seedlings

The quizalofop acid metabolite PPA (95% purity, Yuanye, Shanghai, China) was dissolved in DMSO and diluted with 0.1% (v/v) Tween 80 to the specified use rates. When at the 3‐ to 4‐leaf stage, SC‐S *P. fugax* seedlings was treated with rates of PPA (0, 6.56, 13.13, 26.25 and 52.5 g a.i. ha^−1^) using a 3WP‐2000 mobile sprayer as described in Section ‘Expression levels of *GSTF2* and *GSTF58* genes in *P. fugax* populations’. The same rates of quizalofop‐p‐ethyl and quizalofop acid were also used as controls. Plant survival was recorded 21 DAT.

### Antioxidant activity assays of recombinant PfGSTF2 and PfGSTF58

The antioxidant activity of PfGSTF2 and PfGSTF58 was detected using the disc diffusion assay as previously described (Zhang *et al*., 2013). In brief, 200 μL of *E. coli* culture transformed with pET‐28a (+)/PfGSTF or pET‐28a (+) (OD600 = 1) was evenly distributed on LB solid medium containing 1 mM IPTG and 50 mg L^−1^ kanamycin. The LB plates were then incubated at 37 °C for 1 h. Cumene hydroperoxide (CHP) is often used as a model substance for extracellular ROS induction (Li *et al*., 2021); therefore, the antioxidant activity of recombinant PfGSTF2 and PfGSTF58 was evaluated by CHP treatment. Sterilized filter papers with a diameter of 5 mm were soaked in different concentrations of CHP dissolved in acetone (0, 10, 20, 40 and 60 mM). These soaked filter papers were placed on the surface of the LB plates. After incubation at 37 °C for 48 h, the bacteriostatic areas around the filter paper were recorded. In addition, the GPOX activity of PfGSTF2 and PfGSTF58 was measured using the GPOX activity assay kit (Solarbio, Beijing, China). Each experiment consisted of three biological replicates per treatment, and the experiment was repeated.

### Assays of the content of H_2_O_2_ and MDA and activity of GPOX

The tillering stage (the 5–6 leaf stage) SC‐R and SC‐S plants were treated (using the sprayer) with quizalofop‐p‐ethyl (6.56 g a.i. ha^−1^), and the leaf tissue was collected 72 h after treatment together with untreated plants. Hygromycin‐resistant rice calli were transferred to fresh N6D solid media supplemented with 0 and 10 nM quizalofop‐p‐ethyl, and the rice calli was collected at 72 h after treatment. Histochemical staining with DAB was performed to detect H_2_O_2_ according to a described protocol (Tiwari *et al*., 2022; Xing *et al*., 2022). Briefly, *P. fugax* leaves and rice calli were infiltrated with 10 mM PBS (pH 7.0) containing 1 mg mL^−1^ DAB (Sigma, Shanghai, China) and then incubated at 37 °C in the dark for 5 h. The leaves and rice calli were then rinsed with ultrapure water and transferred to 95% (v/v) ethanol for decolourization for 1 h. the *P. fugax* leaves were photographed with a Canon camera and the rice calli with a stereomicroscope. For the determination of MDA content and GPOX activity, 0.1 g of *P. fugax* leaf tissue or rice calli was frozen in liquid nitrogen and ground to fine powder using a pestle and mortar. MDA content was determined using the MDA content assay kit (Solarbio, Beijing, China), and GPOX activity determined using the GPOX activity assay kit (Solarbio, Beijing, China). Assays of MDA content and GPOX activity of *P. fugax* contained 4 to 5 biological replicates. The MDA content assay of rice calli contained 8 biological replicates and each calli line (0.1 g) as one biological replicate.

### Data analysis

The datasets from repeated experiments were analysed by SPSS v23 (IBM, Armonk, USA) using the general linear model procedure for variance analysis. There was no significant difference between repeated experiments (*P* > 0.05) and the repeated data sets were combined. Significance between means in all comparing data sets was analysed by Student's *t*‐test using the SPSS v23 software.

## Conflicts of interest

The authors declared that they have no conflicts of interest.

## Author contributions

L.P., L.B. and Y.Q. designed and supervised the study. W.C., D.B. and Y.X. collected samples. W.C., D.B. and Y.X. conducted all experiments and validations. W.C. and D.B. performed data curation and visualization. W.C., D.B., Y.Q. and L.P. wrote the manuscript. W.C., D.B., Y.X., Y.Q., L.B. and L.P. discussed the results and edited the manuscript. All authors read and approved the manuscript.

## Supporting information


**Figure S1.** Figure S1. Sequence alignment of the two *GST2c* genes (*GST2c‐1* and *GST2c‐2*) in SC‐R and SC‐S *P. fugax* plants.
**Figure S2.** Phylogenetic relationships and conserved motifs between PfGST2c and the wheat GSTs genome.
**Figure S3.** Lack of difference in herbicide sensitivity of the rice calli expressing *PfGSTF2* and *PfGSTF58* versus *GFP* control.
**Figure S4.** Overexpression of *PfGSTF2* confers quizalofop‐p‐ethyl resistance in rice seedlings.
**Figure S5.** CRISPR/Cas9‐induced *OsGSTF2* (LOC_Os01g27360) gene editing in rice.
**Figure S6.** Growth response to quizalofop‐p‐ethyl of untransformed rice seedlings (WT) vs CRISPR/Cas9 knockout seedlings for the orthologues gene *OsGSTF2 (osgstf2)*.
**Figure S7.** Characterization of *E. coli* recombinant protein PfGSTF2 and PfGSTF58.
**Figure S8.** *In vitro* NBD‐Cl inhibition of *E. coli* expressed PfGSTF2 and PfGSTF58 activity.
**Figure S9.** UHPLC‐Q‐TOF‐MS analysis of *in vitro* quizalofop acid metabolism by *E. coli* expressed PfGSTF58 and PfGSTF2.
**Figure S10.** UHPLC‐Q‐TOF‐MS analysis of herbicide metabolism.
**Figure S11.** Structural features and interactions between PfGSTF2 and ligand.
**Figure S12.** The best docking poses of PfGSTF58 and PfGSTF2 binding to CDNB and quizalofop acid.
**Figure S13.** Antioxidant activity of PfGSTF2 and PfGSTF58.
**Figure S14.** Levels of ROS, MDA and GPOX activity in plants of SC‐R and SC‐S *P. fugax* populations.
**Figure S15.** Sequence alignment of plant phi‐class GSTs and the predicted secondary structure elements of the PfGSTF2.
**Figure S16.** Proposed framework for *PfGSTF2*‐mediated metabolic resistance to quizalofop‐p‐ethyl in *P. fugax*.
**Figure S17.** Construction of heterologous expression cassette of *PfGSTF2* and *PfGSTF58* in *E. coli*.
**Table S1.** Primers used in this study.

## Data Availability

The data that support the findings of this study are available on request from the corresponding author. The data are not publicly available due to privacy or ethical restrictions.
