# Supplementary material for: PfGSTF2 endows resistance to quizalofop‐p‐ethyl in Polypogon fugax by GSH conjugation
Source: Plant Biotechnol J. 2024 Oct 28;23(1):216–31. doi: 10.1111/pbi.14491 (PMC11672750; doi:10.1111/pbi.14491)
Supplement: Supplementary file 1 — Figure S1. Figure S1. Sequence alignment of the two GST2c genes (GST2c‐1 and GST2c‐2) in SC‐R and SC‐S P. fugax plants. Figure S2. Phylogenetic relationships and conserved motifs between PfGST2c and the wheat GSTs genome. Figure S3. Lack of difference in herbicide sensitivity of the rice calli expressing PfGSTF2 and PfGSTF58 versus GFP control. Figure S4. Overexpression of PfGSTF2 confers quizalofop‐p‐ethyl resistance in rice seedlings. Figure S5. CRISPR/Cas9‐induced OsGSTF2 (LOC_Os01g27360) gene editing in rice. Figure S6. Growth response to quizalofop‐p‐ethyl of untransformed rice seedlings (WT) vs CRISPR/Cas9 knockout seedlings for the orthologues gene OsGSTF2 (osgstf2). Figure S7. Characterization of E. coli recombinant protein PfGSTF2 and PfGSTF58. Figure S8. In vitro NBD‐Cl inhibition of E. coli expressed PfGSTF2 and PfGSTF58 activity. Figure S9. UHPLC‐Q‐TOF‐MS analysis of in vitro quizalofop acid metabolism by E. coli expressed PfGSTF58 and PfGSTF2. Figure S10. UHPLC‐Q‐TOF‐MS analysis of herbicide metabolism. Figure S11. Structural features and interactions between PfGSTF2 and ligand. Figure S12. The best docking poses of PfGSTF58 and PfGSTF2 binding to CDNB and quizalofop acid. Figure S13. Antioxidant activity of PfGSTF2 and PfGSTF58. Figure S14. Levels of ROS, MDA and GPOX activity in plants of SC‐R and SC‐S P. fugax populations. Figure S15. Sequence alignment of plant phi‐class GSTs and the predicted secondary structure elements of the PfGSTF2. Figure S16. Proposed framework for PfGSTF2‐mediated metabolic resistance to quizalofop‐p‐ethyl in P. fugax. Figure S17. Construction of heterologous expression cassette of PfGSTF2 and PfGSTF58 in E. coli. Table S1. Primers used in this study. [file PBI-23-216-s001.docx]

**PfGSTF2 endows resistance to** **quizalofop-p-ethyl in *Polypogon fugax* by GSH-conjugation**

Wen Chen^1, 2^, Dingyi Bai^1^, Yuxi Liao^1^, Qin Yu^3,^ *, Lianyang Bai^1,^ * and Lang Pan^1,^ *

^1^ College of Plant Protection, Hunan Agricultural University, Changsha, 410128, China.

^2^ College of Agriculture, Tarim University, Alaer, 843300, China.

^3^Australian Herbicide Resistance Initiative (AHRI), School of Agriculture and Environment, University of Western Australia, Australia, WA 6009.

^*^**Corresponding authors:** L Pan ([langpan@hunau.edu.cn](mailto:langpan@hunau.edu.cn)), LY Bai (lybai@hunaas.cn), Q Yu ([qin.yu@uwa.edu.au](mailto:qin.yu@uwa.edu.au)).

**
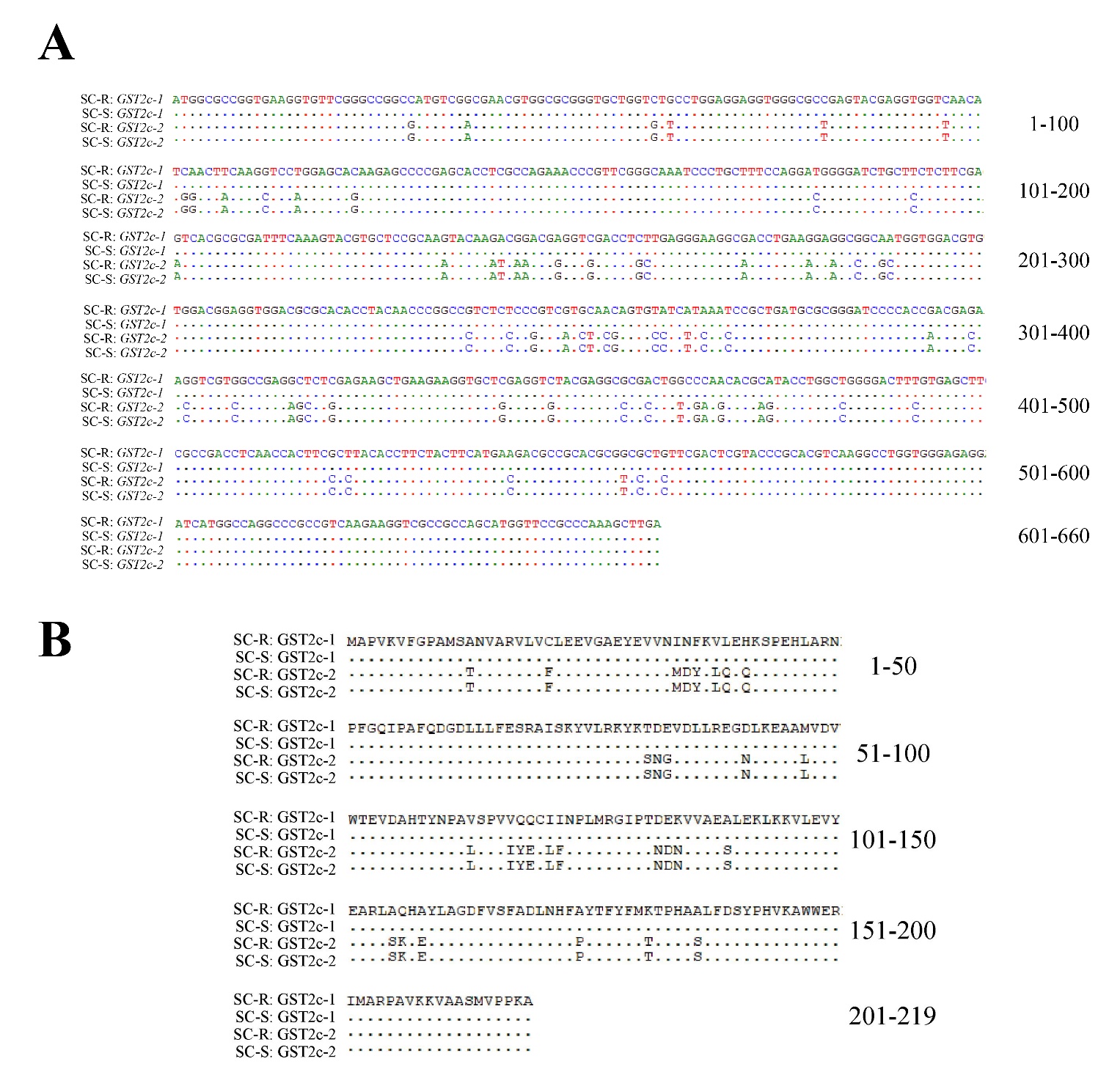
**

**Figure S1.** Sequence alignment of the two *GST2c* genes (*GST2c-1* and *GST2c-2*) in SC-R and SC-S *P. fugax* plants. (A) nucleotide sequences, and (B) deduced amino acid sequences.


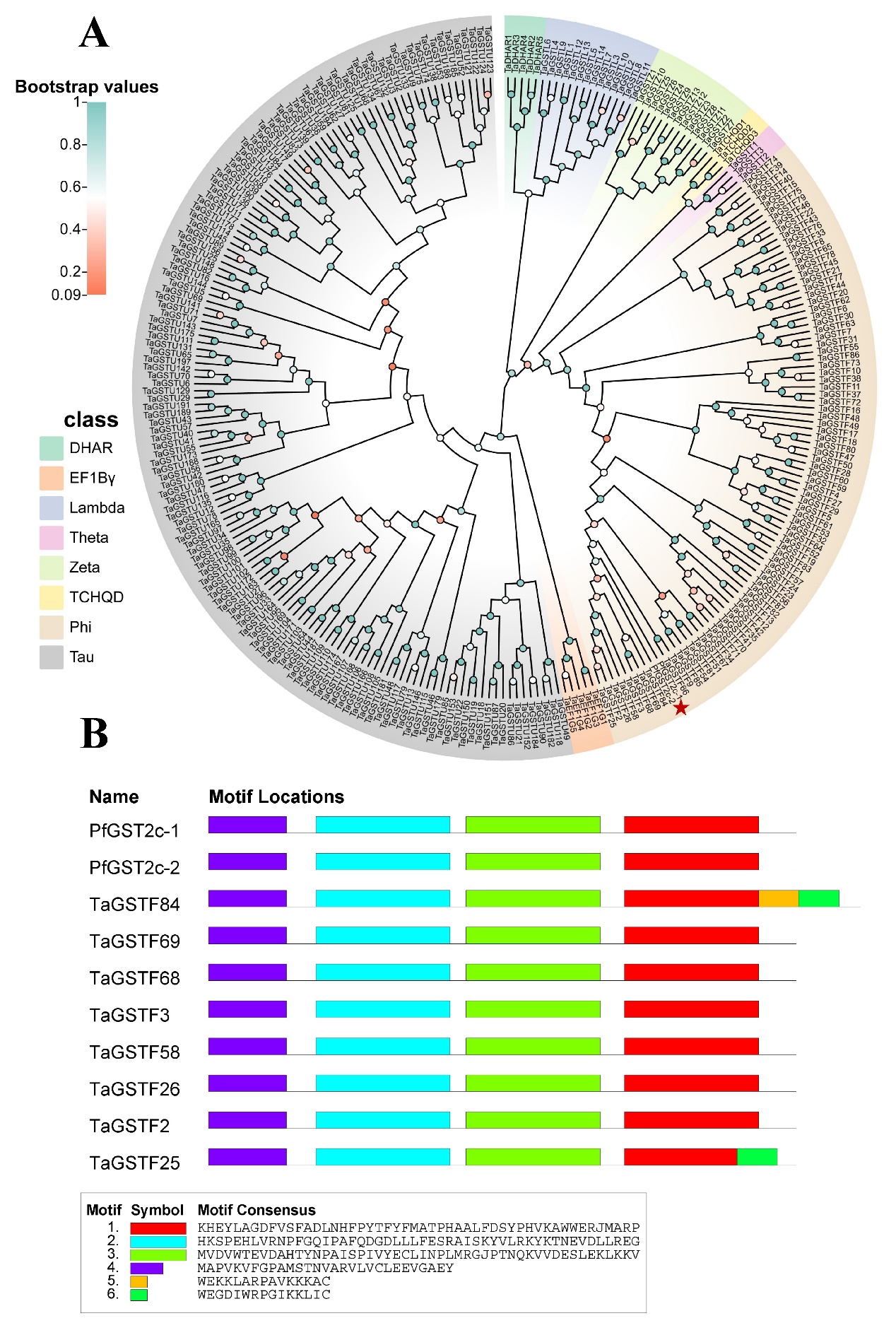


**Figure S2.** Phylogenetic relationships (A) and conserved motifs (B) between PfGST2c and the wheat GSTs genome. The neighbour-joining method was used in the tree reconstruction. Bootstrap values from a sample of 1000 replicates are shown on each branch.


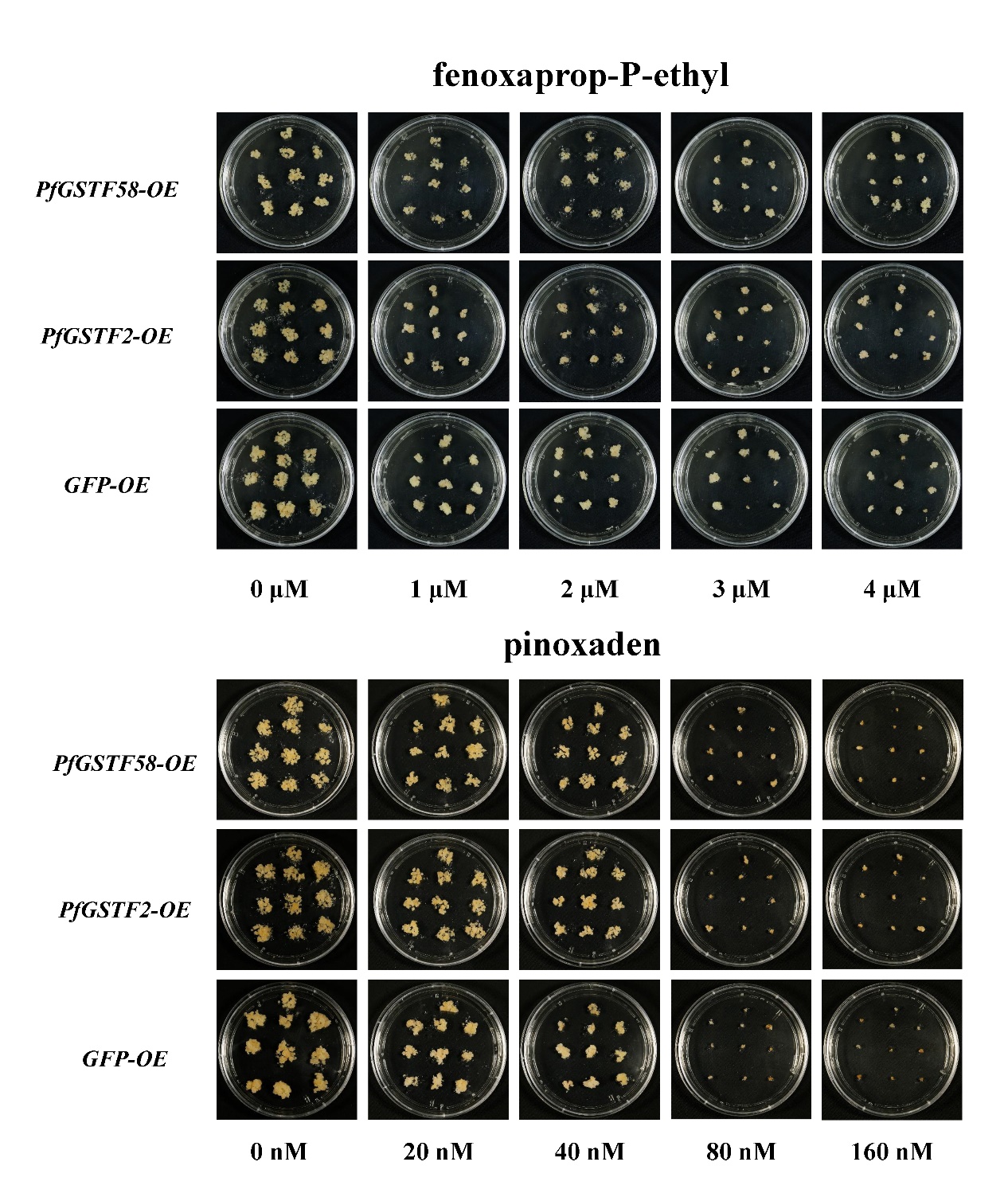


**Figure S3.** Lack of difference in herbicide sensitivity of the rice calli expressing *PfGSTF2* and *PfGSTF58* versus *GFP* control. Ten independent calli expressing *PfGSTF2, PfGSTF58* or *GFP* were selected with hygromycin and grown for 3 weeks on N6D medium.


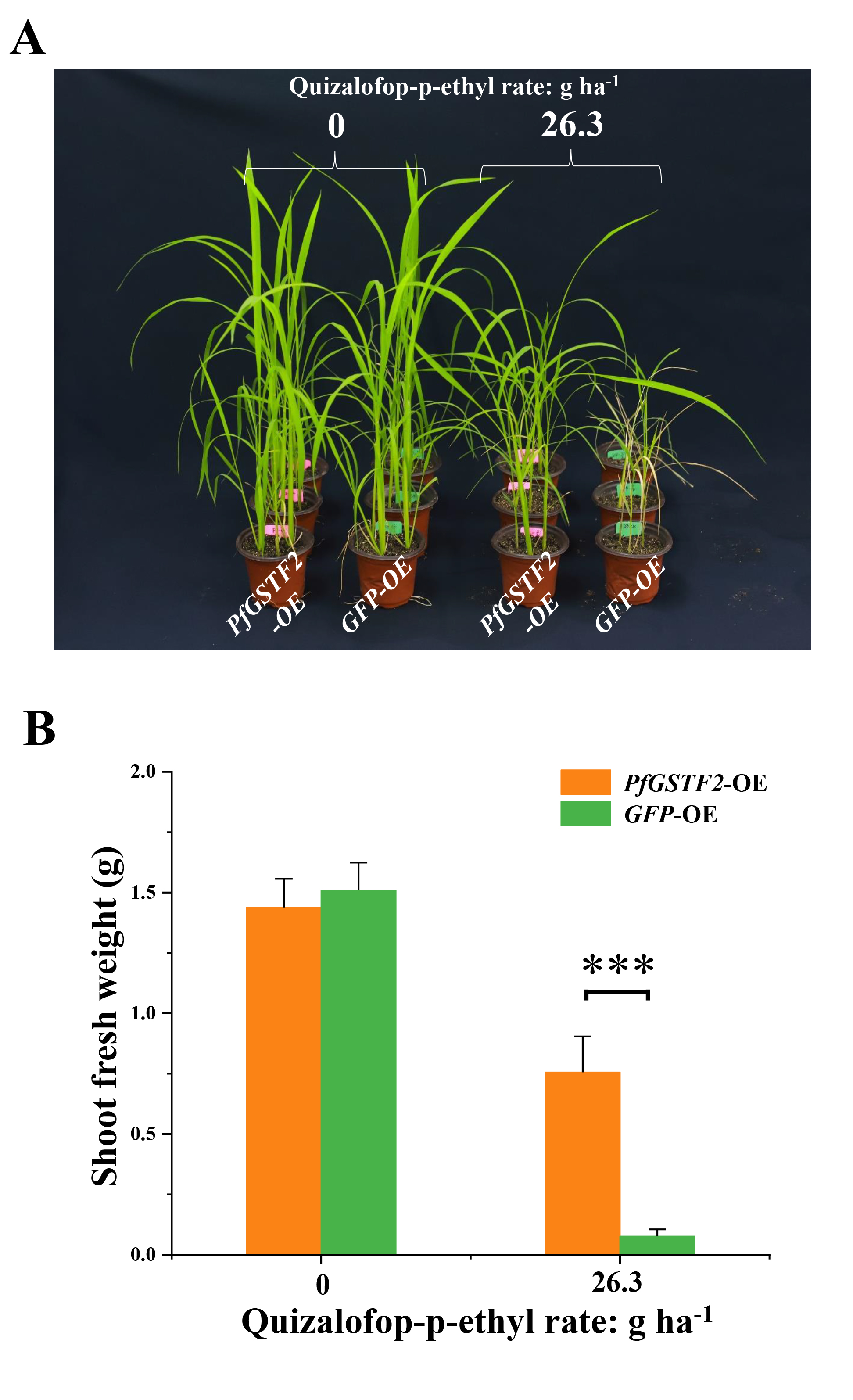


**Figure S4.** Overexpression of *PfGSTF2* confers quizalofop-p-ethyl resistance in rice seedlings. Growth response (A) and shoot fresh weight (B) of rice T_0_ seedlings transformed with the *GFP* (control) or *PfGSTF2* gene, three weeks after quizalofop-p-ethyl treatment. Data are means ± SE (n=9). *** indicates significant difference (*p* < 0.001) by the Student’s *t*-test.


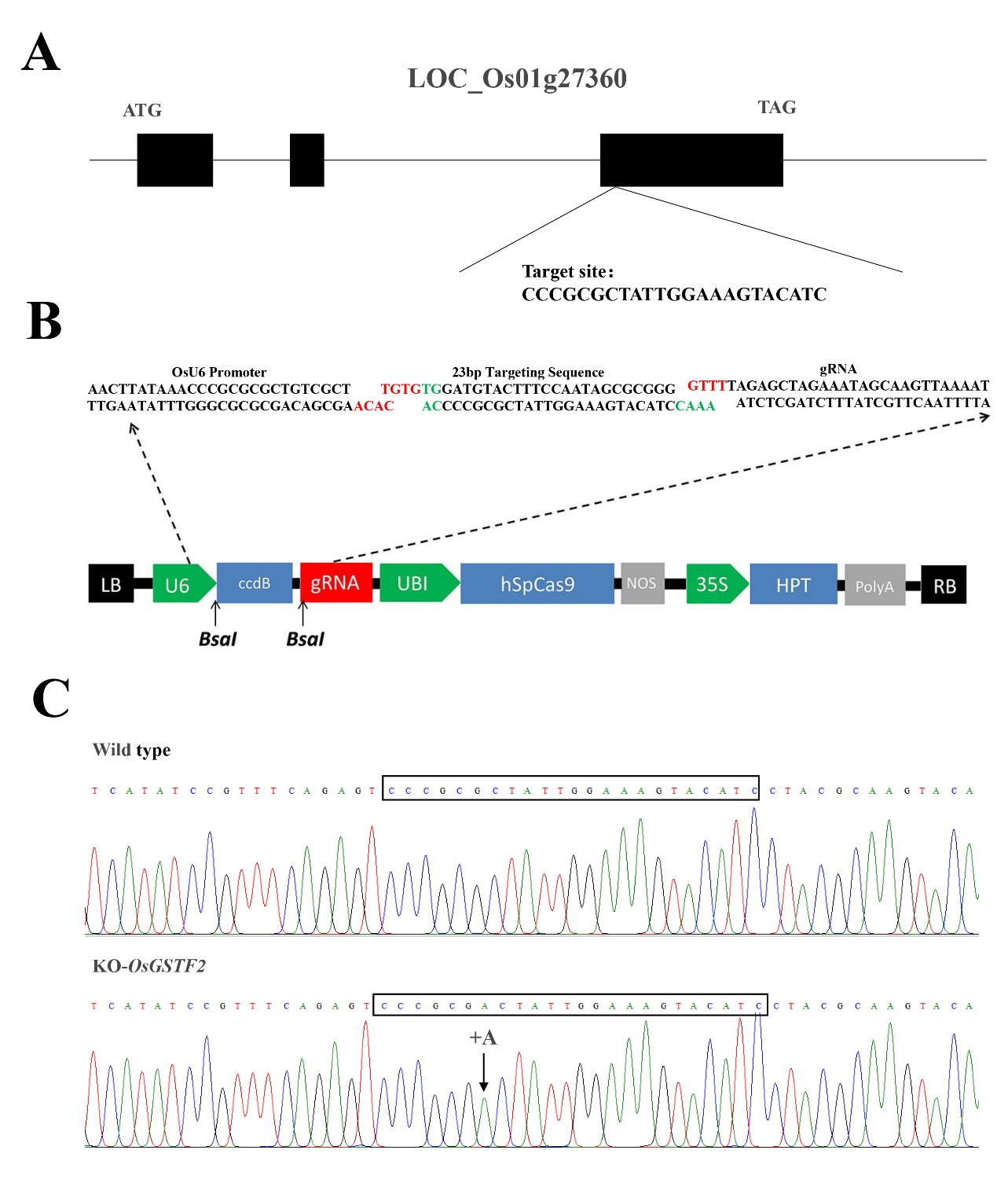


**Figure S5.** CRISPR/Cas9-induced *OsGSTF2* (LOC_Os01g27360) gene editing in rice. (A) Schematic diagram of the LOC_Os01g27360 gene structure and the target site. Exons and introns are indicated with black rectangles and black lines, respectively. (B) Structure of the CRISPR/Cas9 binary vector pBGK032. The key sequences and restriction sites for cloning are given. The expression of Cas9 is driven by the maize ubiquitin promoter (UBI); the expression of the sgRNA scaffold is driven by the rice U6 small nuclear RNA promoter (OsU6), and the expression of hygromycin (HPT) is driven by CaMV35S promoters (35S). Abbreviations: NOS, gene terminator; LB and RB, left border and right border, respectively. (C) Nucleotide sequences at the target site in T_0_ rice mutants. The recovered knockout mutant allele sequence (KO-*OsGSTF2*) was shown below the wild type sequence. Target site nucleotides are in black boxes. The inserted nucleotide is arrowed.


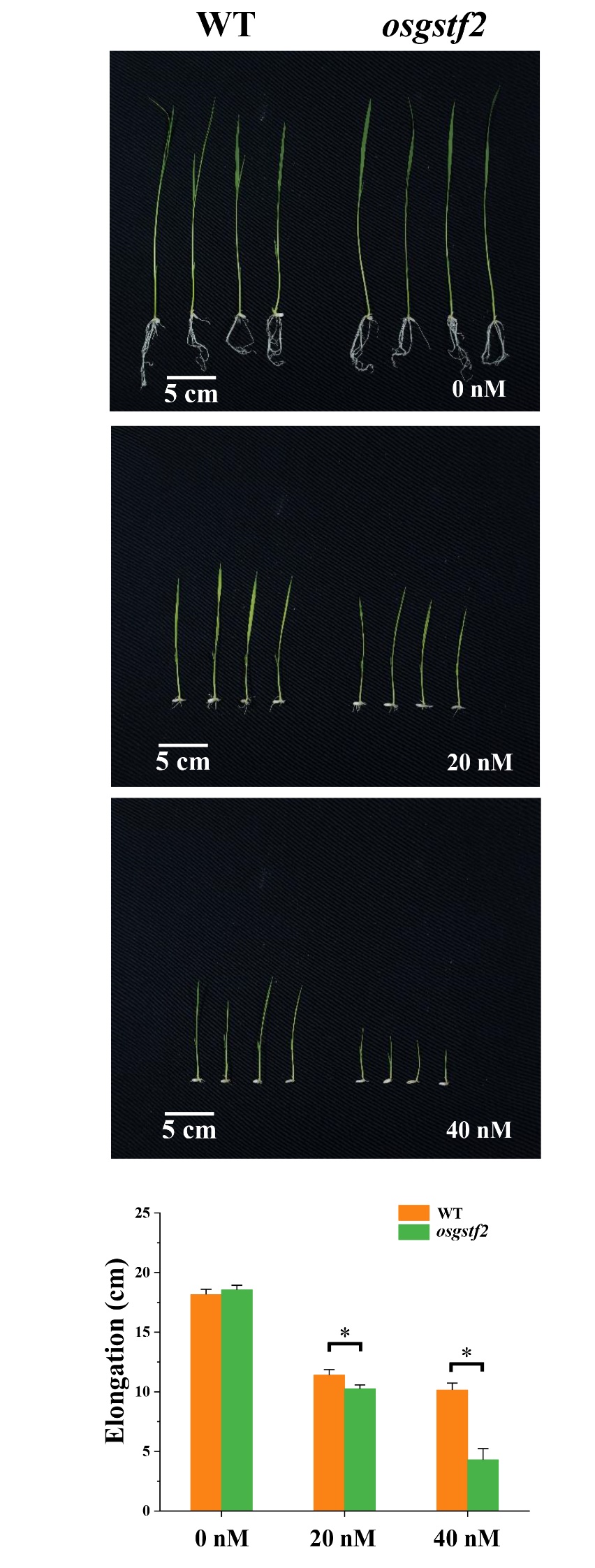


**Figure S6.** Growth response to quizalofop-p-ethyl of untransformed rice seedlings (WT) vs CRISPR/Cas9 knockout seedlings for the orthologues gene *OsGSTF2 (osgstf2).* Seedlings from one line were cultured for 7 d on half strength MS medium supplemented with 0, 20, and 40 nM quizalofop-p-ethyl and shoot elongation recorded. Data are means ± SE (n=6).

**
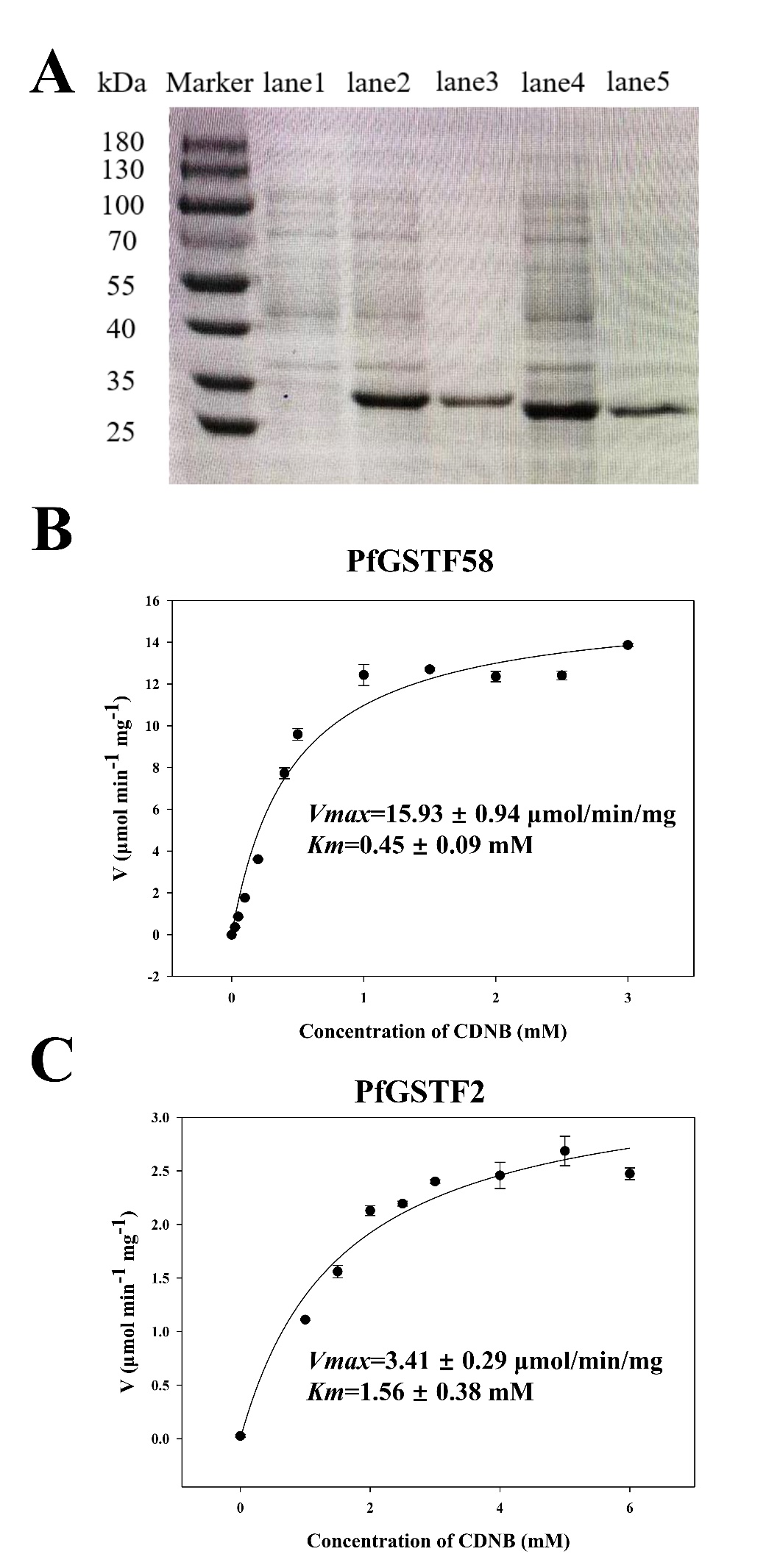
**

**Figure S7.** Characterization of *E. coli* recombinant protein PfGSTF2 and PfGSTF58. (A) SDS-PAGE analysis of molecular mass of recombinant protein of PfGSTF2 and PfGSTF58. Marker: protein marker; Lane 1, crude recombinant protein of pET-28a (+) induced by IPTG; Lane 2, crude recombinant protein of pET-28a (+)/PfGSTF58 induced by IPTG; Lane 3, purified recombinant protein of pET-28a (+)/PfGSTF58 eluted by 200 mM imidazole; Lane 4, crude recombinant protein of pET-28a (+)/ PfGSTF2 induced by IPTG; Lane 5, purified recombinant protein of pET-28a (+)/ PfGSTF2 eluted by 200 mM imidazole. Enzyme kinetic parameters of (B) PfGSTF58 and (C) PfGSTF2 against the standard substrate CDNB, estimated with the Michaelis−Menten plot.


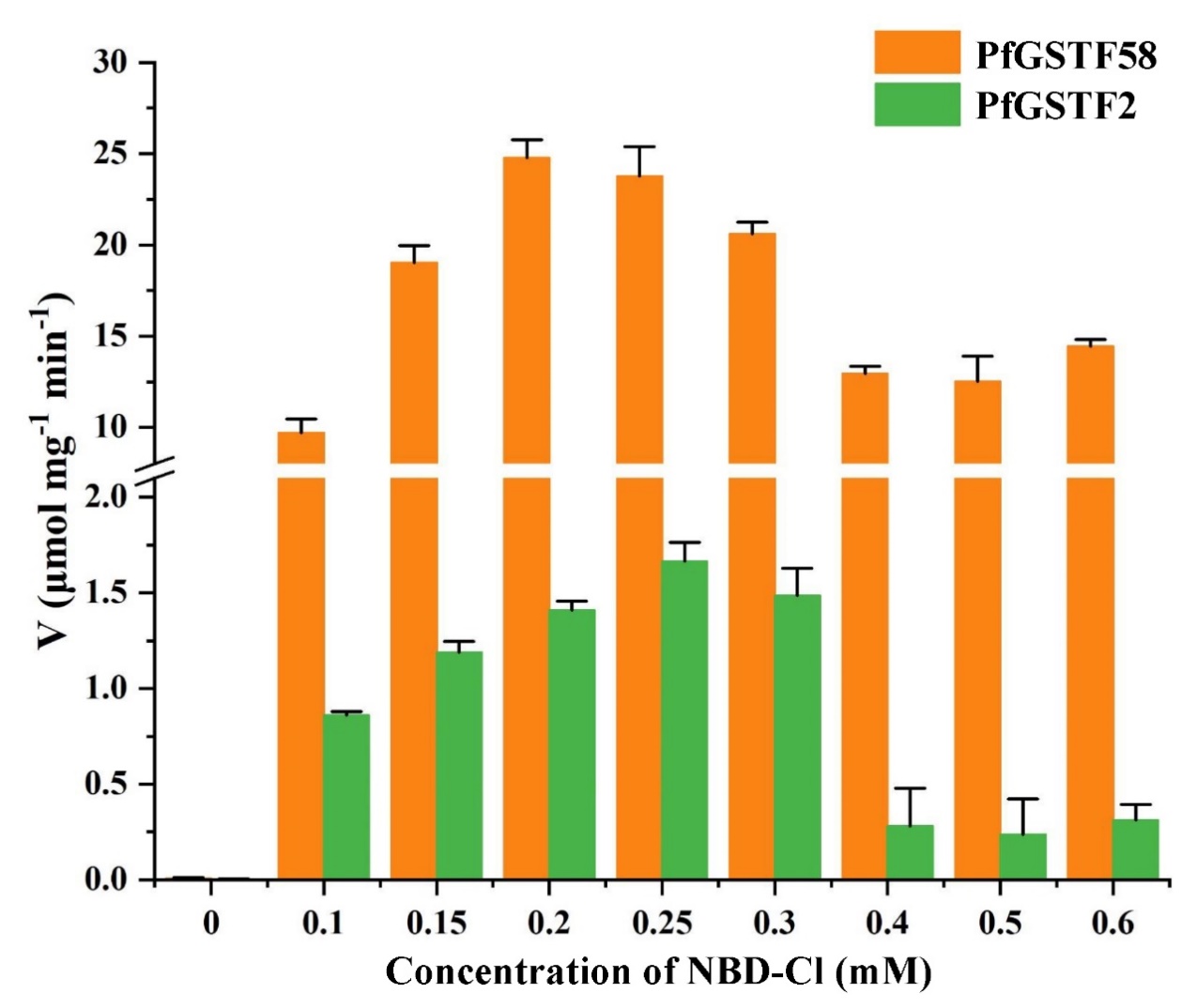


**Figure S8.** *In vitro* NBD-Cl inhibition of *E. coli* expressed PfGSTF2 and PfGSTF58 activity. Data are means ± SE (n=3).


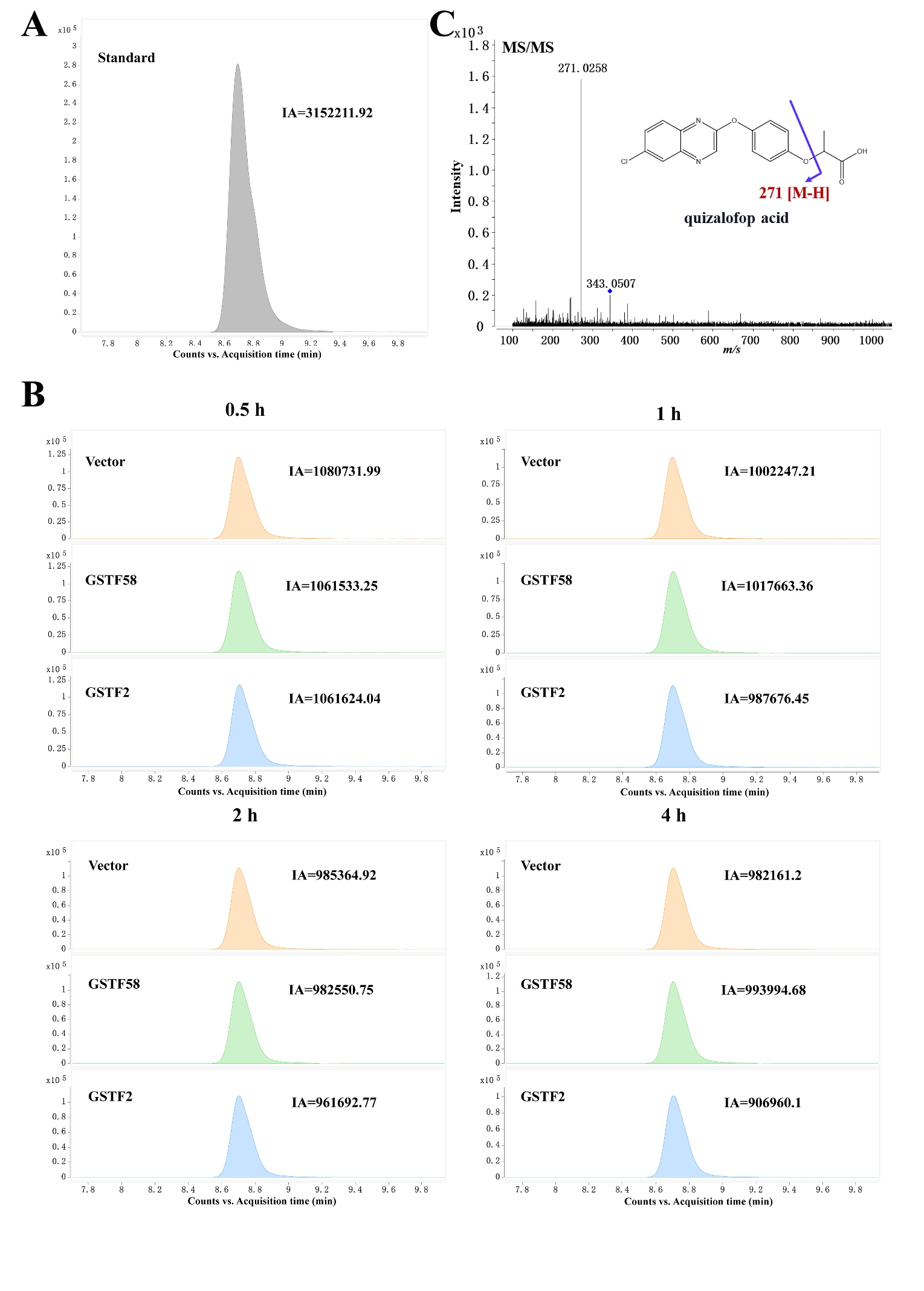


**Figure S9.** UHPLC-Q-TOF-MS analysis of *in vitro* quizalofop acid metabolism by *E. coli* expressed PfGSTF58 and PfGSTF2. (A) Standard analytical grade quizalofop acid. (B) Levels of quizalofop acid remaining in the reaction mixture in comparison to the vector pET28a, 0.5, 1, 2, and 4 h after incubation with glutathione and quizalofop acid. (C) Second-order mass spectrum of quizalofop acid (peak at *m/z* 343.051). Vector: Mixture of pET28a, glutathione and quizalofop acid as a control. IA: integral area.


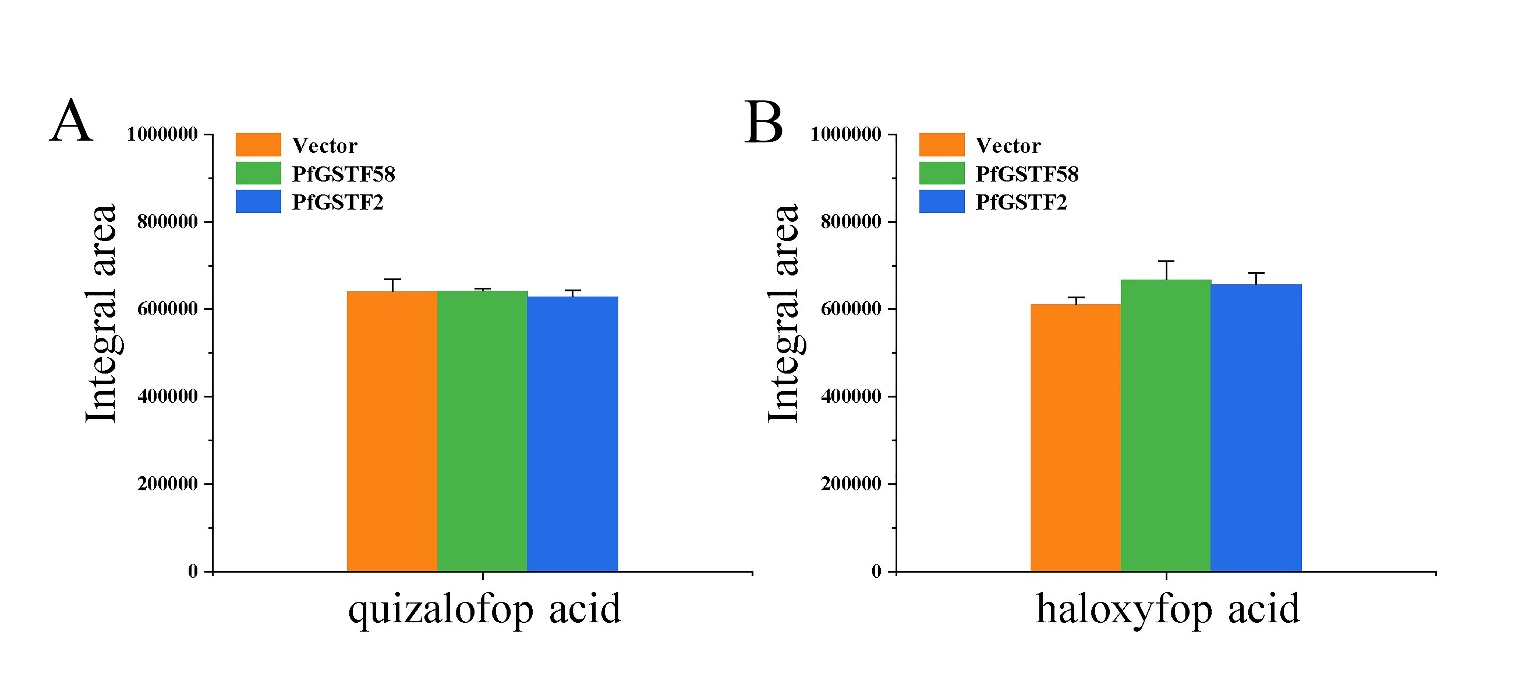


**Figure S10.** UHPLC-Q-TOF-MS analysis of herbicide metabolism. (A) UHPLC-Q-TOF-MS analysis showing lack of *in vitro* metabolism of quizalofop acid (similar levels of the acid) by *E. coli* expressed PfGSTF58 and PfGSTF2 as compared to the vector, 8 h after incubation with quizalofop acid in the absence of GSH. (B) UHPLC-Q-TOF-MS analysis showing lack of *in vitro* metabolism of haloxyfop acid (similar levels of the acid) by *E. coli* expressed PfGSTF58 and PfGSTF2 as compared to the vector, 8 h after incubation with haloxyfop acid in the presence of GSH. Data are means ± SE (n=3).

**
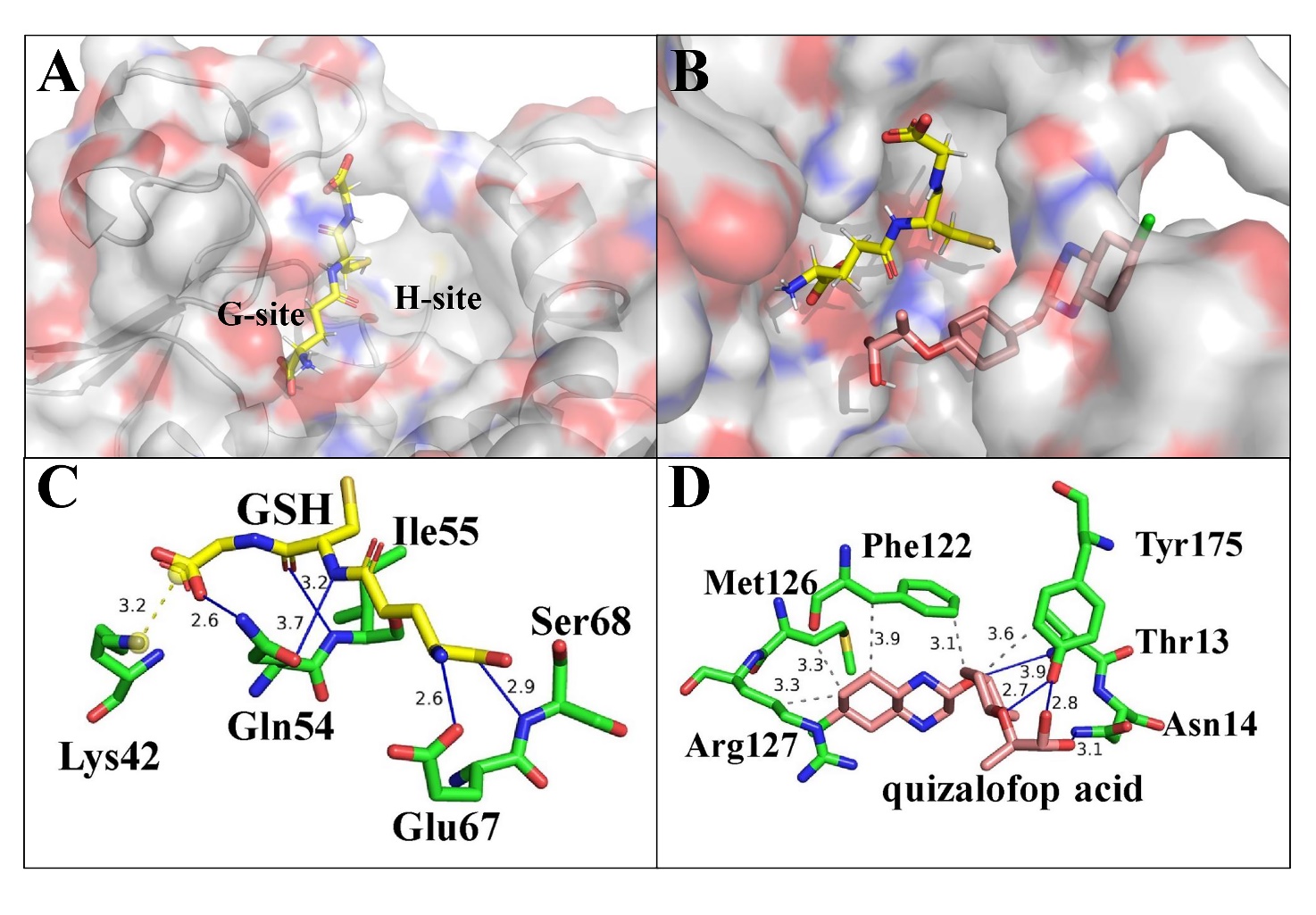
**

**Figure S11.** Structural features and interactions between PfGSTF2 and ligand. (A) Predicted surface representation of GSH binding in PfGSTF2. (B) Predicted surface representation of quizalofop acid binding and PfGSTF2. Predicted residues that may contribute to the interaction of PfGSTF2 with GSH (C) and quizalofop acid (D). GSH, residues and quizalofop acid are represented by the yellow, green and rose red stick, respectively. G-site, GSH binding site; H-site, electrophilic binding site.

**
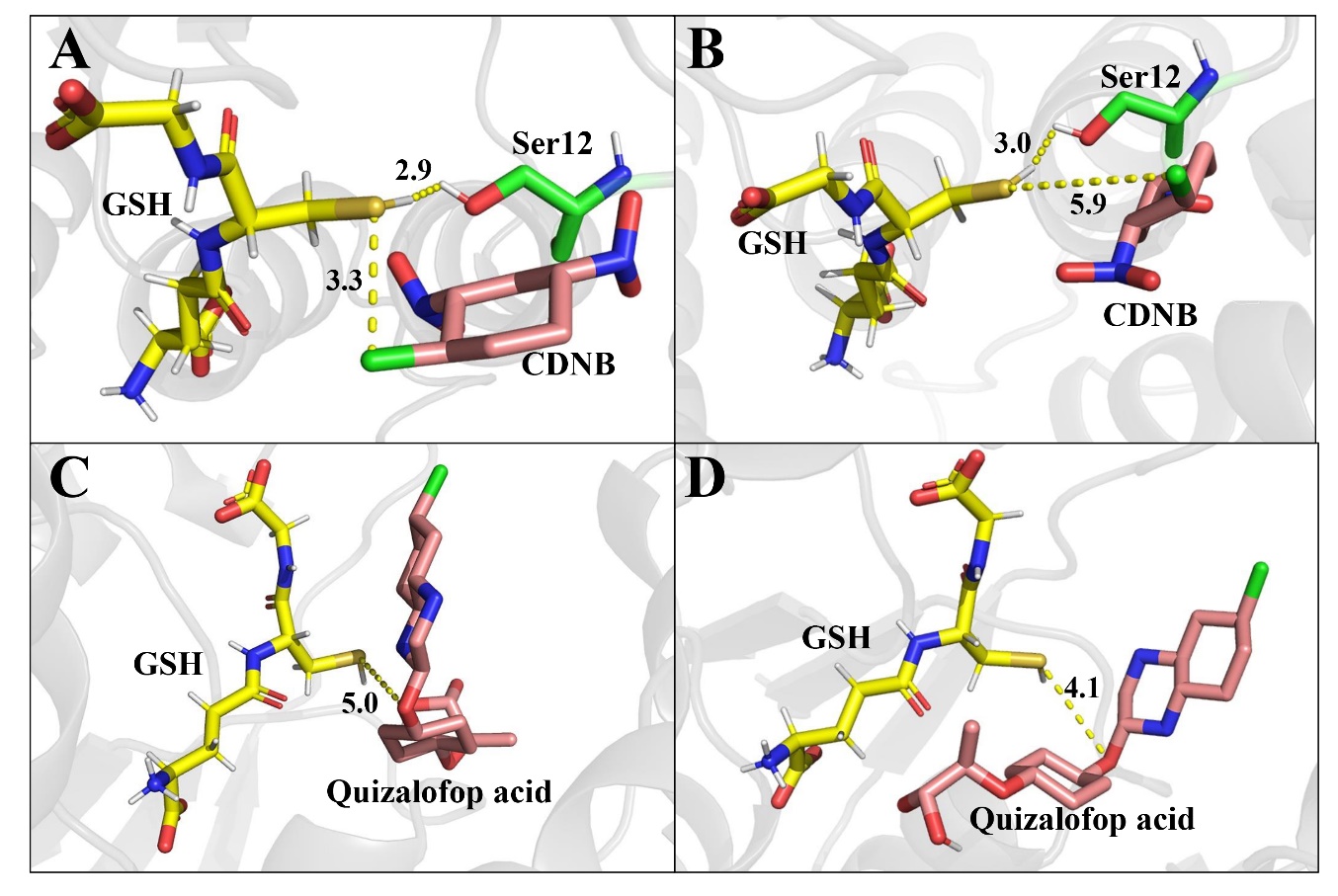
Figure S12.** The best docking poses of PfGSTF58 (A, C) and PfGSTF2 (B, D) binding to CDNB and quizalofop acid. GSH, residues and herbicides are represented by the yellow, green and rose red sticks, respectively. Distance between the sulfhydryl group of GSH and the O atom of the herbicide was expressed in Angstrom and shown by a dotted line.


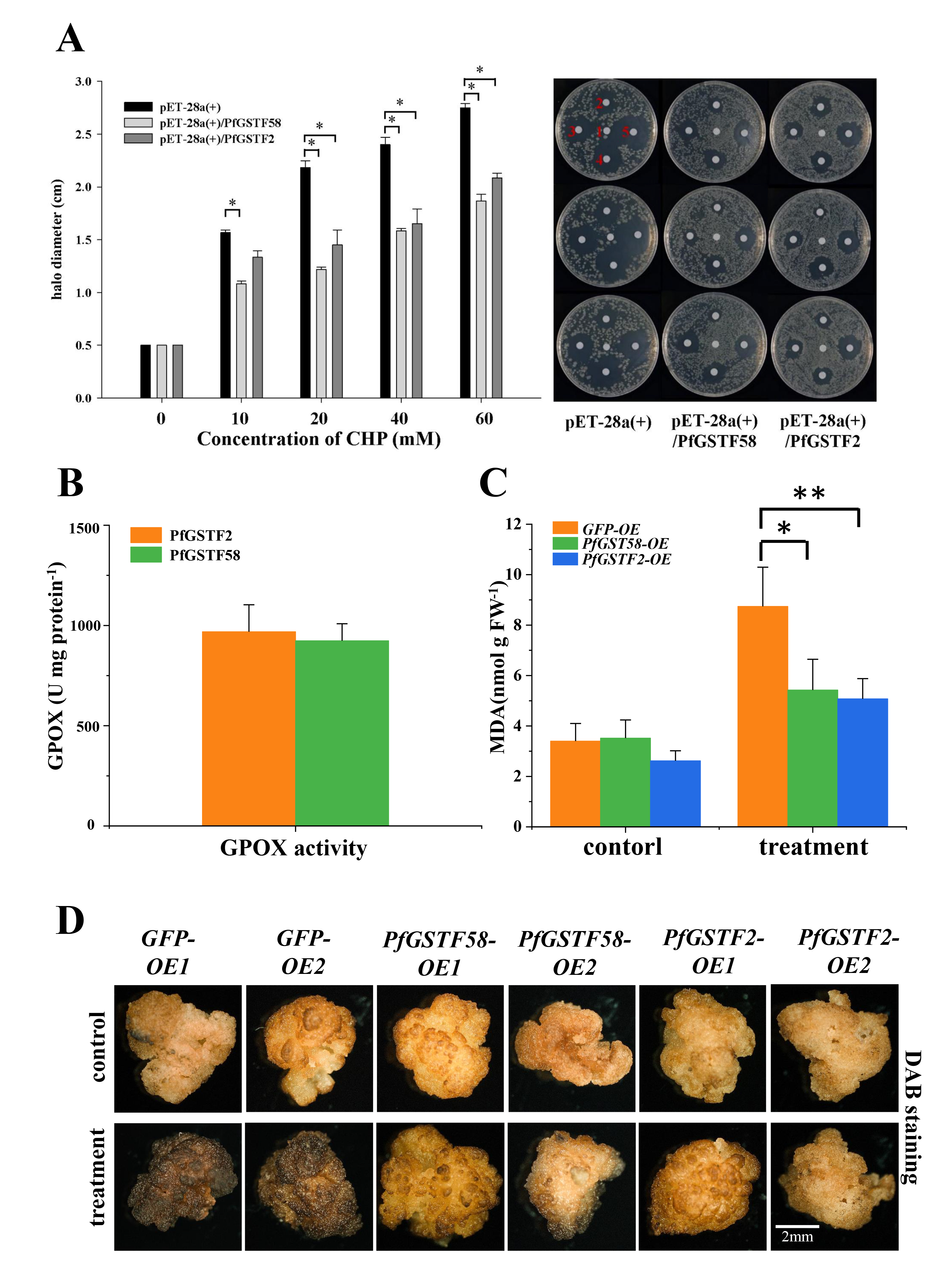


**Figure S13.** Antioxidant activity of PfGSTF2 and PfGSTF58. (A) The halo diameter of *E. coli*-expressed pET-28a (+) and pET-28a (+)/ PfGSTF. Antioxidant activity was measured using a disc diffusion assay against cumene hydroperoxide (CHP). Labels 1−5 in the Petri dish correspond to 0, 10, 20, 40, and 60 mM CHP, respectively (B) GPOX activity of *E. coli* expressed PfGSTF58 and PfGSTF2. (C) MDA content in transgenic *GFP*, *PfGSTF2* and *PfGSTF58* rice calli 3 d after quizalofop-p-ethyl treatment (10 nM). (D) Peroxide detection by DAB staining in transgenic *GFP*, *PfGSTF2* and *PfGSTF58* rice calli, respectively, after the herbicide treatment. Data are means ± SE (A and B, n=3. C, n=8). * and ** indicates significant difference (*p* < 0.05 and 0.01, respectively) between the vector control and OE, by the Student’s *t*-test.


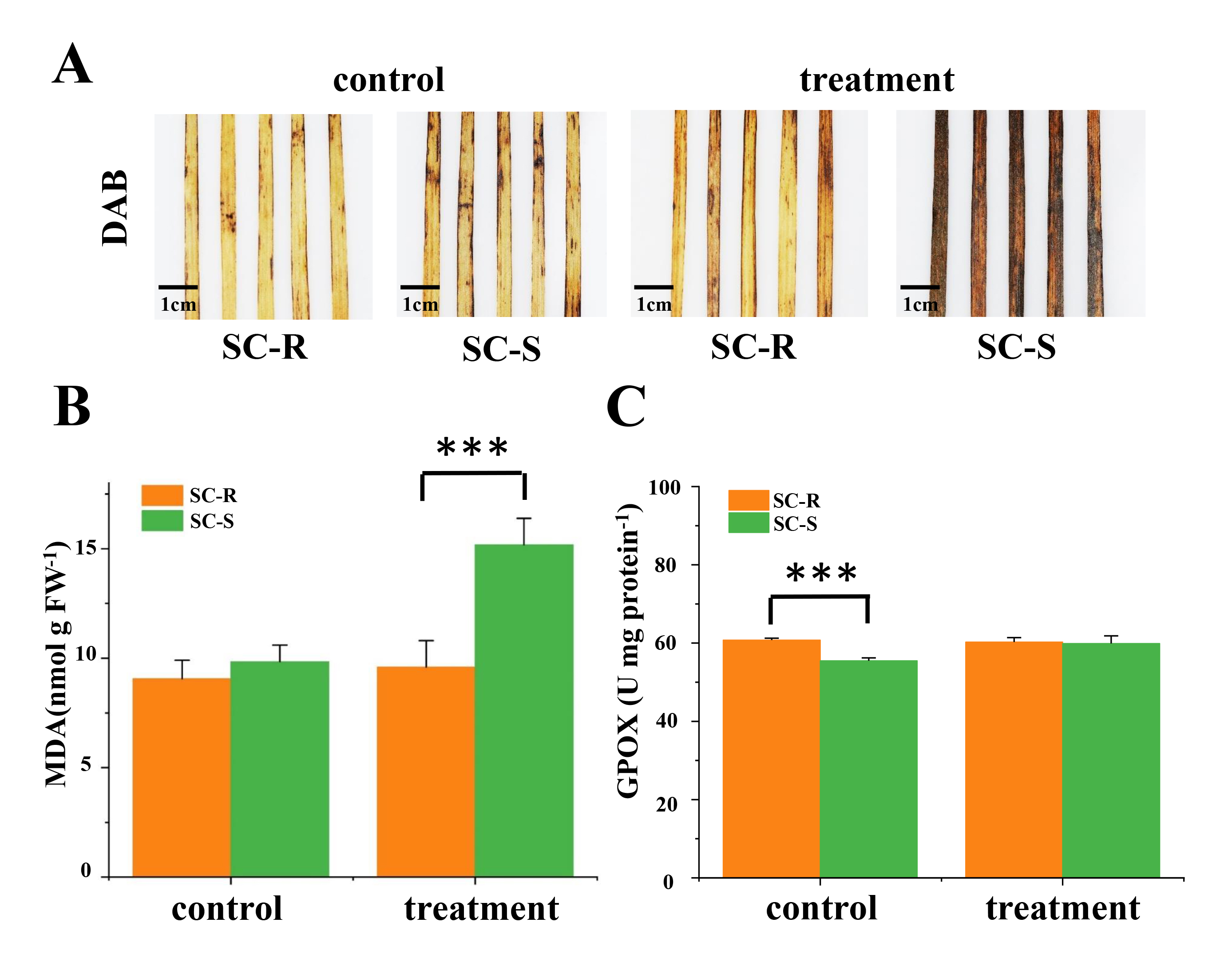


**Figure S14.** Levels of reactive oxygen species (A), malondialdehyde (MDA, B) and GPOX activity (C) in plants of SC-R and SC-S *P.* *fugax* populations, 3 d after quizalofop-p-ethyl treatment (6.56 g a.i. ha^-1^). Peroxide was detected by DAB staining. Data are means ± SE (B, n=4. C, n=5). *** indicates *p* < 0.001, by the Student’s *t*-test.


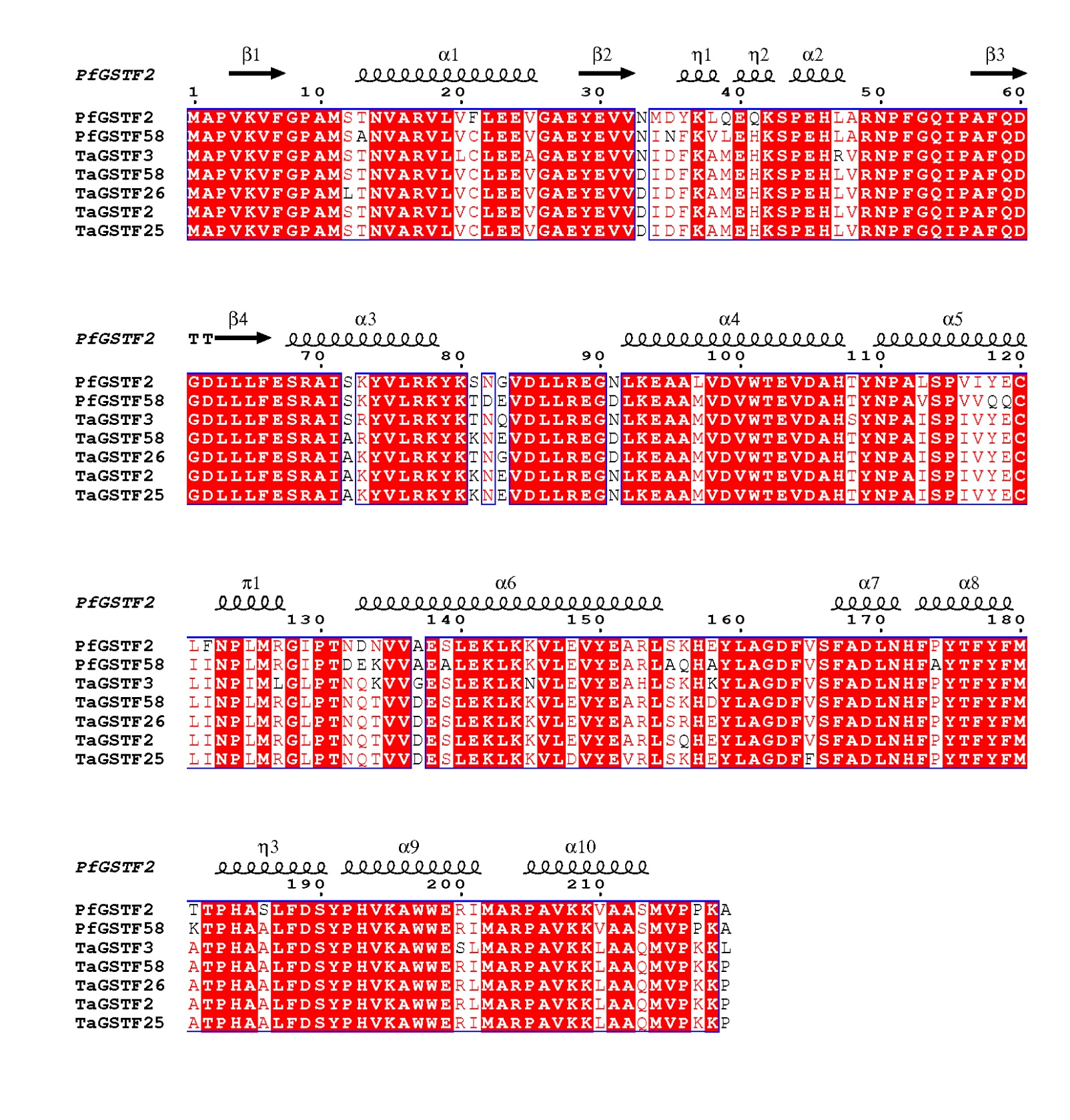
**Figure S15.** Sequence alignment of plant phi-class GSTs and the predicted secondary structure elements of the PfGSTF2. Alpha helices and beta strands are represented as helices and arrows, respectively. Conserved residues in plant phi-class GSTs are in red block.

Photos were taken 3 weeks after treatment. Data are means ± SE of three biological replicates per treatment. * indicates significant difference (*p* < 0.05) by the Student’s *t*-test.

**
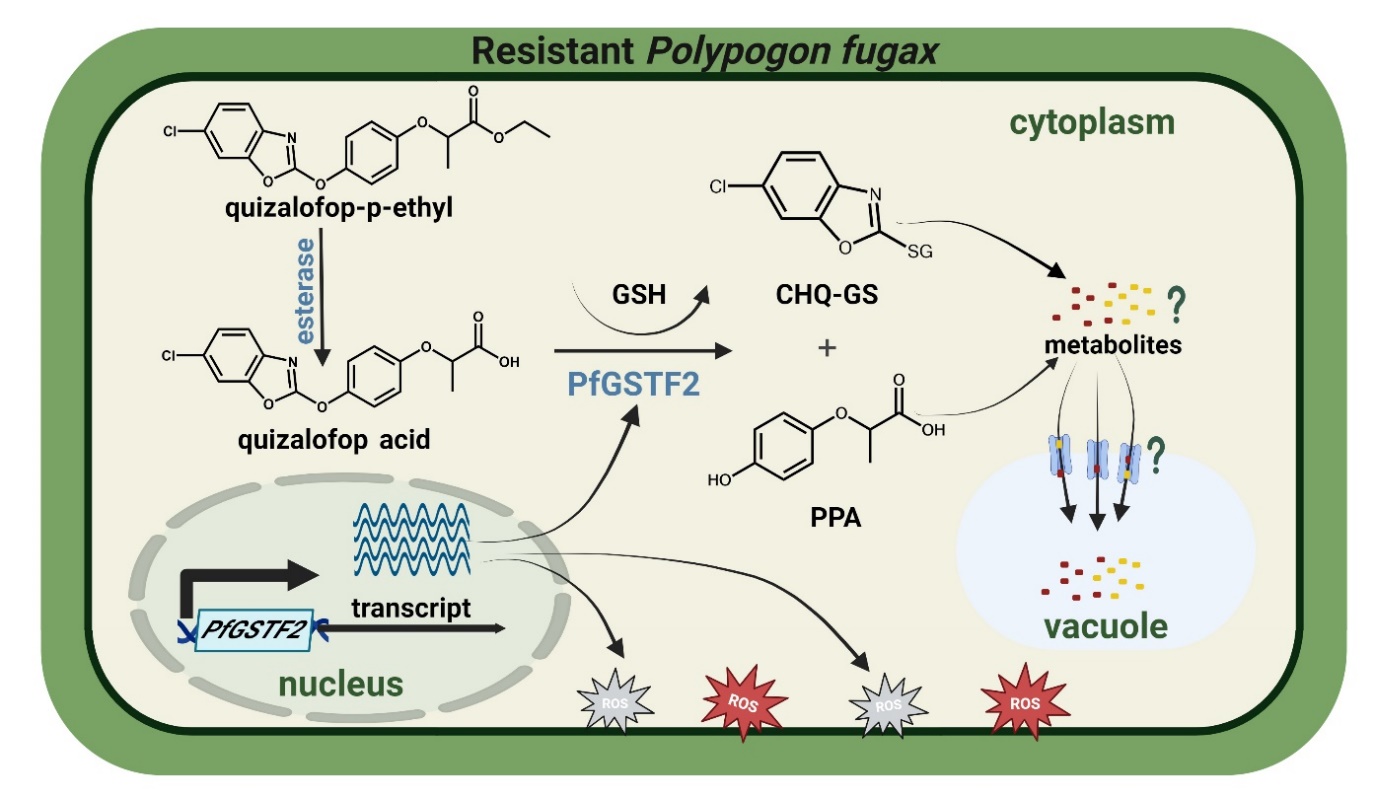
Figure S16.** Proposed framework for *PfGSTF2*-mediated metabolic resistance to quizalofop-p-ethyl in *P. fugax*. In the resistant plants, *PfGSF2* gene expression was enhanced, resulting in higher activity of PfGSTF2 and thus accelerating conjugation of quizalofop acid to form more water soluble and less phytotoxic 6-chloroquinoxalin-2-ol-glutathione (CHQ-GS) conjugate and 2-(4-hydroxyphenoxy) propanoic acid (PPA). CHQ-GS and PPA may undergo further metabolism or are directly moved to vacuoles for compartmentation. In addition, PfGSTF2 may have some antioxidant GPOX activity, reducing reactive oxygen species (ROS) accumulation, thereby alleviating cytotoxicity.


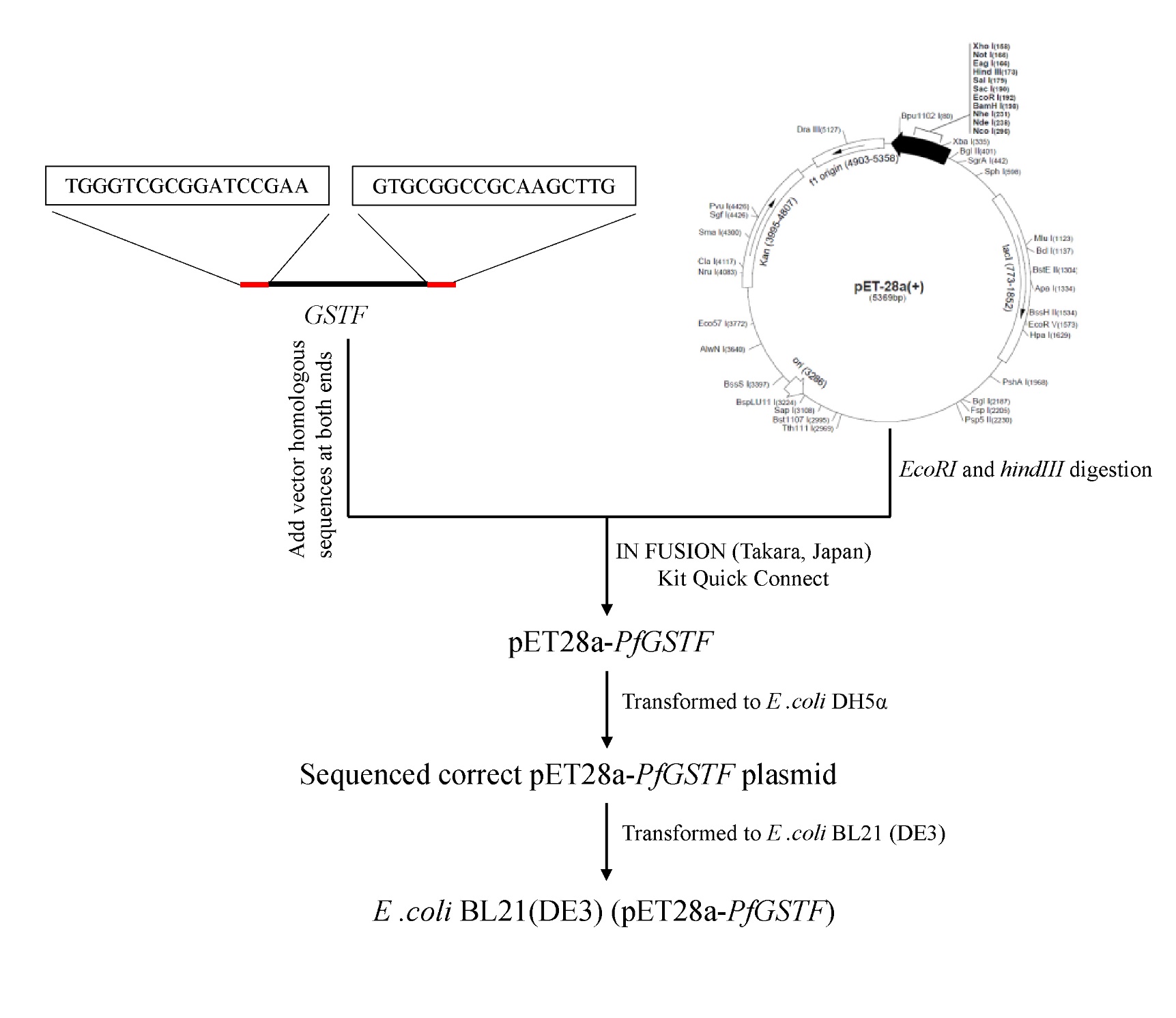
**Figure S17.** Construction of heterologous expression cassette of *PfGSTF2* and *PfGSTF58* in *E. coli*.

**Table S1.** Primers used in this study.

| Primer | | Direction | Sequence (5′–3′) | Usage |
| --- | --- | --- | --- | --- |
| PfGST2c-full | Forward | ATGGCGCCGGTGAAGGTGTT | Full-length *PfGST2c* cDNA amplification |  |
|  | Reverse | TCAMGCCTTKGGCGGAACCATG |  |  |
| PfGSTF58-Q | Forward | AGGCGACCTGAAGGAGGCGGCAAT | RT-qPCR |  |
|  | Reverse | GGTATGCGTGTTGGGCCAGT |  |  |
| PfGSTF2-Q | Forward | ATGGACTACAAGCTCCAGGAGCAG |  |  |
|  | Reverse | CCACCCCGTTTGATTTGTATTTGC |  |  |
| M13 | Forward | GGTAACGCCAGGGTTTTCC | Clone sequencing |  |
|  | Reverse | CAGGAAACAGCTATGACC |  |  |
| GSTF-POX | Forward | TGTTACTTCTGCAGGATGGCGCCGGTGAAGGTGTT | Full-length *PfGSTF2* and *PfGSTF58* cDNA amplification for In Fusion ligation with POX vector |  |
|  | Reverse | CGGATCCATAACGCGTCAAGCTTTGGGCGGAACCA |  |  |
| GSTF-pET28a | Forward | TCGCGGATCCGAATTCATGGCGCCGGTGAAGGTG | Full-length *PfGSTF2* and *PfGSTF58* cDNA amplification for In Fusion ligation with pET28a vector |  |
|  | Reverse | GTGCGGCCGCAAGCTTGTCAAGCTTTGGGCGGAACCATGCT |  |  |
